# Characterization of the *Giardia intestinalis* secretome during interaction with human intestinal epithelial cells: The impact on host cells

**DOI:** 10.1371/journal.pntd.0006120

**Published:** 2017-12-11

**Authors:** Showgy Y. Ma’ayeh, Jingyi Liu, Dimitra Peirasmaki, Katarina Hörnaeus, Sara Bergström Lind, Manfred Grabherr, Jonas Bergquist, Staffan G. Svärd

**Affiliations:** 1 Department of Cell and Molecular Biology, Uppsala University, BMC, Uppsala, Sweden; 2 Department of Chemistry-BMC, Analytical Chemistry, Uppsala University, Uppsala, Sweden; 3 Department of Medical Biochemsitry and Microbiology, BMC, Uppsala, Sweden; McGill university, CANADA

## Abstract

**Background:**

*Giardia intestinalis* is a non-invasive protozoan parasite that causes giardiasis in humans, the most common form of parasite-induced diarrhea. Disease mechanisms are not completely defined and very few virulence factors are known.

**Methodology:**

To identify putative virulence factors and elucidate mechanistic pathways leading to disease, we have used proteomics to identify the major excretory-secretory products (ESPs) when *Giardia* trophozoites of WB and GS isolates (assemblages A and B, respectively) interact with intestinal epithelial cells (IECs) *in vitro*.

**Findings:**

The main parts of the IEC and parasite secretomes are constitutively released proteins, the majority of which are associated with metabolism but several proteins are released in response to their interaction (87 and 41 WB and GS proteins, respectively, 76 and 45 human proteins in response to the respective isolates). In parasitized IECs, the secretome profile indicated effects on the cell actin cytoskeleton and the induction of immune responses whereas that of *Giardia* showed anti-oxidation, proteolysis (protease-associated) and induction of encystation responses. The *Giardia* secretome also contained immunodominant and glycosylated proteins as well as new candidate virulence factors and assemblage-specific differences were identified. A minor part of *Giardia* ESPs had signal peptides (29% for both isolates) and extracellular vesicles were detected in the ESPs fractions, suggesting alternative secretory pathways. Microscopic analyses showed ESPs binding to IECs and partial internalization. Parasite ESPs reduced ERK1/2 and P38 phosphorylation and NF-κB nuclear translocation. *Giardia* ESPs altered gene expression in IECs, with a transcriptional profile indicating recruitment of immune cells via chemokines, disturbances in glucose homeostasis, cholesterol and lipid metabolism, cell cycle and induction of apoptosis.

**Conclusions:**

This is the first study identifying *Giardia* ESPs and evaluating their effects on IECs. It highlights the importance of host and parasite ESPs during interactions and reveals the intricate cellular responses that can explain disease mechanisms and attenuated inflammatory responses during giardiasis.

## Author summary

*Giardia intestinalis* is a major contributor to the enormous burden of diarrheal diseases with 280 million symptomatic human infections (giardiasis) per year, mainly in children. Nonetheless, there is poor insight into how *Giardia* causes disease; it is not invasive and very few virulence factors are known. Here we use proteomics, large-scale protein identification, to identify proteins released by the parasite and host cells when they interact *in vitro*. Our analyses show that both cell types respond to interaction by the release of specific proteins. These proteins affect the metabolism of host cells and parasites, and many are involved in the modification of host’s immune responses. The secreted parasite proteins bind to host cells and some are internalized, resulting in the modulation of immune-signaling proteins, cell cycle arrest and the induction of cell death. This study provides new insights into host-parasite interactions during giardiasis and this will be the basis for further studies of specific proteins that might explain disease induction and symptoms during giardiasis.

## Introduction

*Giardia intestinalis* is an intestinal protozoan parasite causing 280 million human diarrheal cases (giardiasis) annually [1]. *Giardia* has two life-cycle stages: infectious cysts and disease-causing trophozoites [2]. Trophozoites move by flagella and adhere to intestinal epithelial cells (IECs) strongly using a suction cup on the ventral surface [2]. The direct contact of *Giardia* with IECs induces cellular damage on the apical surface, compromising brush border enzymes function, and affect cellular junctions, leading to an increased intestinal barrier permeability [3]. These changes cause maldigestion, malabsorption and electrolyte imbalance, collectively culminating in causing diarrhea [4]. Symptoms are usually acute but chronicity is not uncommon and some patients develop intestinal and extra-intestinal complications post-infection [1,5]. Many *Giardia* infections, however, remain asymptomatic and the reason for variable disease outcomes remains obscure.

Early studies of *Giardia* pathogenicity suggested that the parasite releases proteins that can contribute to disease induction. Excretory-secretory products (ESPs) of *Giardia* affect intestinal absorption and secretion [6], cause histopathological alterations [7] and induce specific antibodies against glycoproteins [8]. A secreted 58 kDa glycoprotein has been shown to react with antibodies from giardiasis patients and induce fluid accumulation in ligated rabbit ileal loop experiments [9,10]. Nevertheless, the identity of this protein remained unknown as tryptic digests did not align with any proteins in the *Giardia* Database. Cysteine proteases (CPs) are also yet unidentified *Giardia* ESPs. Their involvement in virulence, however, is well studied, demonstrated by their association with parasite attachment [11], and the degradation of extracellular matrix (ECM) proteins [12], antibodies [13] and proinflammatory cytokines [14,15]. Arginine metabolising enzymes (AMEs) (e.g. arginine deiminase (ADI) and ornithine carbamoyltransferase (OCT)) are the best-studied examples of secreted virulence factors in *Giardia*. AMEs outcompete host cells for arginine uptake, inhibiting cellular proliferation [16], abrogating nitric oxide production, a compound cytotoxic to *Giardia*, [17], and inhibiting cytokine production by dendritic cells [18]. Other reports documented the secretion of elongation factor-1 alpha (EF-1α) [19] and the glycolytic enzyme enolase [17] into *Giardia* growth medium and the medium of interaction with IECs *in vitro*, respectively. Their functions in parasite virulence, if any, is still unknown. Finally, variable surface proteins (VSPs) are cysteine-rich proteins that cover trophozoite surface and they are released in large quantities into the growth medium (≈70% within 24h) [20]. VSPs are immunodominant during infection [21] and their constant switching (i.e. antigenic variation) help the parasite evade the host immune system [22].

In view of the simple endomembrane system of *Giardia*, which lacks a true Golgi apparatus, peroxisomes and endosomal/lysosomal system [2], the secretion of proteins, their sorting and trafficking remain a perplexing aspect of the parasite’s biology. Most of our knowledge about secretion and secreted proteins of *Giardia* derive from experiments targeting the sorting and trafficking of VSPs to the plasma membrane and cyst-wall proteins in encystation-specific vesicles (ESVs) to the cyst wall during encystation [23]. AMEs, EF-1α and enolase all lack signal peptides (SPPs) but they are still secreted by the parasite. This suggests the presence of non-conventional secretory mechanisms in *Giardia* such as extracellular vesicles (EVs) carrying protein cargo with no SSP. EVs include the exosomes (30–100 nm) and microvesicles (MVs) (100–1000 nm), which have been shown to play roles in parasites communication, host cell colonization and immune modulation [24,25]. A recent study has identified MVs released from *Giardia* trophozoites and encysting cells [26]. The released MVs affected trophozoite attachment and dendritic cell activation [26].

In this study, we used proteomics to identify the secretome of the *Giardia* reference isolates WB and GS during growth and interaction with IECs *in vitro* in serum-free medium. We also show the release of EVs by *Giardia* as a part of its secretome. We demonstrate that *Giardia* ESPs alter gene expression in IECs, cell signalling and the production of proinflammatory cytokines. This study highlights the importance of *Giardia* ESPs in inducing pathological effects in IECs and suggests how this might have immunomodulatory effects on host immune responses.

## Materials and methods

### Ethics statement and chemicals

The cell lines used in this study were all obtained from American Type Culture Collection (ATCC): *Giardia intestinalis* isolates WB clone C6 (ATCC30957; assemblage A) and GS, clone H7 (ATCC50581; assemblage B) were used in this study in combination with the human colonic adenocarcinoma cell line, Caco-2 clone TC7 (ATCC HTB-37). All the experiments were approved by the Programme Review Board at Department of Cell and Molecular Biology, Uppsala University, Sweden. All chemicals were purchased from Sigma-Aldrich (MO, USA), unless otherwise stated.

### Parasites and intestinal epithelial cells (IECs)

*Giardia* trophozoites were cultured in 10 ml and 50 ml tubes filled with TYDK medium (37°C) supplemented with 10% heat inactivated bovine serum (Sigma-Aldrich) as described in [27]. The Caco-2 clone TC7 (passage numbers 7–12) cell line was grown and differentiated as previously described [16].

### Medium for collection of excretory/secretory products (ESPs) and trophozoite viability

To collect *Giardia* ESPs from axenic cultures we used a modified RPMI-1640 medium containing 55.5 mM glucose, 22.8 mM L-arginine, 11.4 mM L-cysteine hydrochloride monohydrate, 11.4 mM ascorbic acid, 2mM Glutamax (Gibco), 1 mM sodium pyruvate (Gibco) and 1x MEM essential amino acids. The medium was filter-sterilized and placed in a water bath (final pH is 6.85 at 37°C) until used.

Confluent *Giardia* culture tubes, prepared as described above, were used for trophozoite viability assessment. Briefly, the TYDK medium was decanted from each tube followed by three washes in Hank’s Balanced Salt Solution with glucose (HBSS-G) supplemented with 11.4 mM L-cysteine (pH 6.8, 37°C). Culture tubes were replenished with the modified medium, closed tightly and incubated for 2h or 6h (37°C). At the end of each incubation, tubes were placed on ice to detach trophozoites (10 min), followed by counting using the expulsion of Trypan Blue and flagellar motility as viability criteria.

### Collection and processing of ESPs from axenic cultures and host parasite interactions

**Fig 1:**
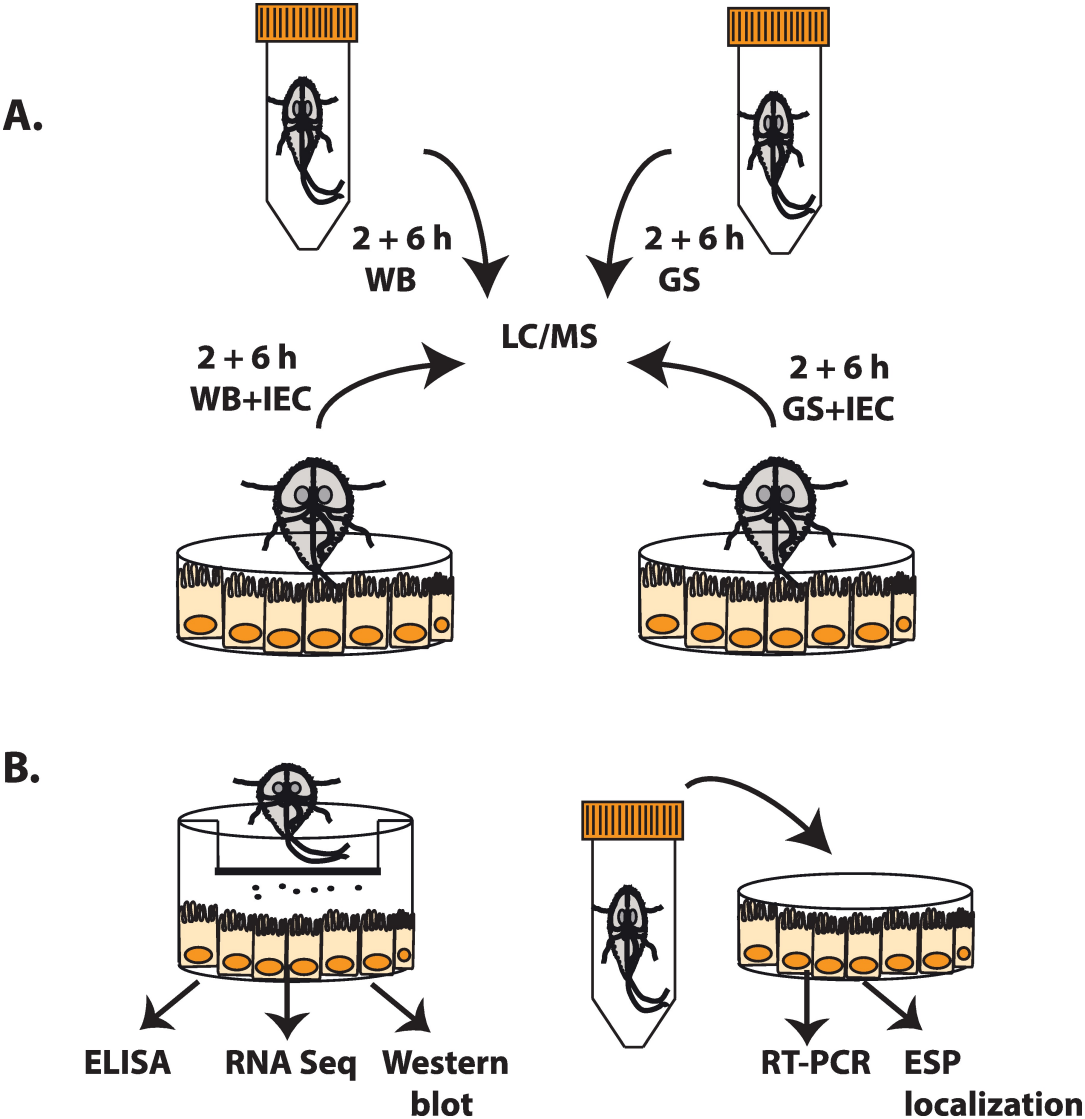
Experimental setup for proteomic analyses and characterization of *Giardia* ESPs. (A) The collection of *Giardia intestinalis*, isolates WB and GS, excretory-secretory products (ESPs) in a serum-free medium from axenic culture and co-culture with differentiated intestinal epithelial cells (IECs), Caco-2, for 2h and 6h. Samples were processed as described in Materials and Methods followed by liquid chromatography/mass spectrometry to identify secreted proteins. (B) Studies of IECs exposed to *G*. *intestinalis* ESPs using differential gene expression analyses by RNA sequencing, quantitative real time PCR, Western blots, ELISA and confocal microscopy. The panel illustrates the experimental setup (left to right) showing IECs exposed to ESPs of either *Giardia* isolates through a Transwell insert (direct exposure) or different amounts of ESPs collected from axenic culture (i.e. the absence of IECs, 1 μg, 5 μg or 10 μg per ml of medium). Abbreviation: Dulbecco’s Modified Eagles Medium (DMEM).

Tubes with *Giardia* trophozoites in modified RPMI-1640 medium (50 ml, two tubes of each isolate) were used to collect *Giardia* ESPs in axenic cultures. The tubes and media, following counting and viability assessment, were centrifuged (930 x*g*, 10 min, 4°C) to pellet trophozoites. Collected media were filter-sterilized (0.22 μM) and treated with protease inhibitor cocktail tablets (cOmplete, Mini EDTA-free, Roche, Sigma-Aldrich). Media were concentrated down to 200–300 μl using the Amicon Ultra 15 mL centrifugal filters with 3kDa cutoff (Merck-Millipore, Darmstadt, Germany). To further eliminate any traces of residual serum, concentrates were processed through the AlbuminOUT kit (G-Biosciences, MO, USA) according to the manufacturer’s instructions. Three biological replicates (2h and 6h) were prepared and stored at -80°C until used for MS analysis. The whole experimental setup is described in Fig 1A.

Differentiated Caco-2 cells (75 cm^2^ flasks) were washed with warm PBS (3x) and incubated with 6 x 10^7^ trophozoites resuspended in 25 ml of DMEM (no FBS) for 2h or 6h. Trophozoites were washed (3x, HBSS-G plus 11.4 mM L-cysteine) prior addition to cells. Caco-2 cells treated as above and incubated alone in DMEM (2h and 6h) served as controls (Fig 1A). All incubations were performed in 10% CO_2_ humidified tissue culture incubator at 37°C. At the end of each interaction, flasks were placed on ice (10 min) to detach trophozoites for counting and viability assessment. Media were then collected, processed as described earlier and stored at -80°C until used for MS analysis. Three biological replicates were prepared (2h or 6h including controls).

### Isolation of *Giardia* extracellular vesicles (EVs) and electron microscopy

The ESP enrichment experiment for axenic cultures described earlier was repeated to collect *Giardia* EVs. Samples were processed with the ExoQuick TC Kit (SBI System Biosciences, CA, USA) according to the manufacturer’s instructions. *Giardia* EVs were pelleted by centrifugation (1500 x*g*, 30 min) and resuspended in 100 μl PBS buffer and processed immediately for electron microscopy (EM). Samples (5μl) were adsorbed onto a Formvar-coated 200 mesh grid (15 seconds), followed by treatment with 2% molybdenum (10 seconds) and contrasting using 2% uranyl acetate (10 seconds). Grids were dried and visualized using a transmission electron microscope at the BioVis Core Facility, Uppsala University, Uppsala, Sweden. A negative control (media alone) was included and processed similarly.

### Liquid chromatography and mass spectrometry (LC/MS) analysis

Before digestion, the buffer in the collected samples was exchanged to a urea-containing lysis buffer (20 mM HEPES, 9 M urea, Complete Mini EDTA-free protease inhibitor cocktail (Roche) using 3 kDa molecular weight cut off filters (Thermo Fisher Scientific, MA, USA). Proteins were then subjected to dithiothreitol (DTT, 12.5 mM) reduction and iodoacetamide (IAA, 6.9 mM) alkylation before digested in-solution with trypsin (1 μg added per sample). The samples were desalted using Pierce C_18_ Spin Columns (Thermo Fisher Scientific). Eluates were dried and resolved in 15 μL 0.1% formic acid of which 5 μL were injected. The nanoLC-MS/MS experiments were performed using a Q Exactive Orbitrap mass spectrometer (ThermoFisher Scientific, Bremen, Germany) equipped with a nano-electrospray ion source. The peptides were separated by C_18_ reversed phase liquid chromatography using an EASY-nLC 1000 system (Thermo Fisher Scientific). A set-up of pre-column and analytical column was used. The pre-column was a 2 cm EASYcolumn (ID 100 μm, 5 μm particles) (Thermo Fisher Scientific) while the analytical column was a 10 cm EASY-column (ID 75 μm, 3 μm particles, Thermo Fisher Scientific). Peptides were eluted with a 150-min linear gradient from 4% to 100% acetonitrile at 250 nL min-1. The mass spectrometer was operated in positive ion mode acquiring a survey mass spectrum with resolving power 70,000 (full width half maximum), m/z 400–1750 using an automatic gain control (AGC) target of 3×10^6^. The 10 most intense ions were selected for higher-energy collisional dissociation (HCD) fragmentation (25% normalized collision energy) and MS/MS spectra were generated with an AGC target of 5×10^5^ at a resolution of 17,500. Mass spectrometer worked in data-dependent mode.

Database searches were performed using the Sequest algorithm, embedded in Proteome Discoverer 1.4 (Thermo Fisher Scientific). Proteins in axenic cultures were identified by performing separate searches against the fasta files of the *Giardia* proteome for each isolate (GS clone containing 4470 entries and WB clone 9667 entries) (available at www.giardiadb.org; giardiadb.org/common/downloads/release5.0/). The fasta file for human proteins was downloaded from Uniprot 2015–05 and contained 20192 entries. Proteins in co-cultures were searched against a combined database of *Giardia* and human proteins in a MudPIT search combining all three replicates. For both searches, the following parameters were used for data processing: Maximum 10 ppm and 0.02 Da error tolerances for the survey scan and MS/MS analysis, respectively, trypsin as digesting enzyme, carbamidomethylation of cysteins as fixed modifications, oxidation of methionine and deamidation of asparagine and glutamine as variable modifications, maximum of two miss cleavages sites. The target decoy PSM validator was used to calculate false discovery rate. The search criteria for protein identification were set to at least two matching peptides of 95% confidence level per protein.

Identified proteins were subject to gene ontology (GO) enrichment analysis to identify their biological as well as proteins class. The analysis was performed using the PANTHER algorithms provided within the Gene Ontology Consortium website (http://geneontology.org/page/go-enrichment-analysis). The results were processed through the GO slim option for biological GO terms, with Bonferroni correction applied for multiple testing (*P* < 0.05). In addition, the STRING Database (http://string-db.org/) was also used to search for InterPro domains, applying Bonferroni correction at *P* < 0.05 as above. Identified proteins were also analyzed for the presence of secretion signal peptides (SSPs) using the SignalP program embedded within the *Giardia*DB (http://giardiadb.org/giardiadb/queries_tools.jsp). The search criteria were set to include any of the following; a SignalP-NN conclusion score of 3, SignalP-NN D-score of 0.5 and SignalP-HMM signal probability of 0.5.

### Differential gene transcription of differentiated Caco-2 cells in response to *Giardia* ESPs

We used RNA sequencing (RNA Seq) or quantitative PCR (qPCR) to identify differential gene expression on the RNA level in IECs (i.e. differentiated Caco-2 cells) exposed to *Giardia* ESPs in two different experimental setups (Fig 1B). First, IECs (6-well plate) were exposed to parasite ESPs via a Transwell insert (0.4 μm pore polyester membrane, Corning, Sigma Aldrich). Here, IECs were physically separated from parasite but directly exposed to their ESPs. For this experiment, we used RNA Seq to identify global changes in gene expression. Second, IECs were exposed to 1, 5 or 10 μg/ml of ESPs from axenic cultures. This aimed at assessing changes in pro-inflammatory gene expression in IECs exposed to ESPs released in axenic culture (i.e. no IECs) and assess how IECs respond to different amounts of *Giardia* ESPs. Changes of gene expression were studied using qPCR. An illustration of the experiments above is depicted in Fig 1B.

The experiments below were performed using complete DMEM with 10% HI-FBS and all incubations were performed in a 10% CO_2_ humidified incubator at 37°C. Before the beginning of all experiments, tissue culture flasks or well-plates were washed twice with warm PBS and incubated with fresh media for 2h. Trophozoite cultures (50 ml) were washed once with warm PBS then cold PBS, incubated on ice (10 min), counted and pelleted by centrifugation (930 x*g*, 10 min, 4°C). Media collected in the insert experiment were centrifuged (930 x*g*, 10 min, 4°C), filter-sterilized (0.45 μM) and stored at -20°C to be used later for cytokines measurement. Three biological replicates were analyzed with RNA Seq for the insert experiment, whereas four biological replicates were used for qPCR in the other experiment. Each biological replicate had a control (IECs alone in media) and IECs exposed to ESPs of either WB or GS isolate. For RNA collection, IECs were lyzed directly in the tissue culture flasks or well-plates using Trizol reagent (Ambion, Thermo Fisher Scientific) and stored at -80°C until processed later.

In the insert experiment, IECs were washed and replenished with 4 ml of fresh DMEM added directly into the well. Trophozoites were processed as stated above and resuspended in DMEM, from which 2 ml (1 x 10^7^ trophozoites) were added into the Transwell inserts and incubated for 2h or 6h. Media were collected at the end of each time point. In the second experiment, IECs (25 cm^2^ flasks) were washed and replenished with 10 ml of fresh DMEM. The amounts of protein in ESPs from either the WB and GS isolates were quantified using the Qubit protein kit (Thermo Fisher Scientific) and added to flasks at a concentration of 1, 5 or 10 μg/ml followed by incubation for 2 h or 6h.

### Enzyme linked immunosorbent assay (ELISA) for cytokines in collected media

Media collected from Caco-2-trophozoite interactions were analyzed by ELISA to measure the concentration of cytokines released by IECs in response to parasite ESPs. The Human CXCL8/IL-8 Quantikine ELISA Kit, Human CCL20/MIP-3 alpha Quantikine ELISA Kit and Human CXCL1/GRO alpha Quantikine ELISA Kit were purchased from R&D Signalling and the samples were processed for cytokine measurements following the manufacturer’s recommendations. The results were plotted and analyzed using a four Parameter Logistic ELISA curve fitting provided at http://www.elisaanalysis.com.

### RNA extraction, library preparation and RNA sequencing

Samples collected as described earlier were processed using the PureLink RNA Mini Kit (Ambion, Thermo Fisher scientific) according to the manufacturer’s instruction. During RNA extraction, a DNaseI treatment step (PureLink DNase Set, Ambion, Thermo Fisher Scientific) was performed on the spin columns to remove genomic DNA. The quality of extracted RNA was assessed by checking the 260/280 and 260/230 ratios using a NanoDrop 1000 Spectrometer (Thermo Fisher Scientific) and by running samples (500 ng) on a 1.5% Tris-Borate-EDTA (TBE) agarose gel prepared with 20 mM of guanidium isothiocyanate (GITC). For cDNA library preparation and RNA Sequencing, RNA (50 ng each) from each sample was reverse transcribed according to the instructions provided in the Ion AmpliSeq Transcriptome Human Gene Expression kit (Revision A.0, Thermo Fisher Scientific) and the generated cDNA was amplified using the Ion AmpliSeq Transcriptome Human Gene Expression core panel (Thermo Fisher Scientific). Primer sequences were partially digested followed by adaptors ligation (Ion P1 Adapter and Ion Xpress Barcode Adapter, Thermo Fisher Scientific) and purification using the Agencourt AMPure XP reagent (Beckman Coulter Inc., CA, USA). Adaptor-ligated amplicons were eluted into the amplification mix (Platinum PCR SuperMix High Fidelity and Library Amplification Primer Mix, Thermo Fisher Scientific) and amplified. Amplicons were subject to size-selection and purification using Agencourt AMPure XP reagent (Beckman Coulter) and were quantified using the Fragment Analyzer instrument (Advanced Analytical Technologies Inc., Ankeny, IA, USA) with DNF-474 High Sensitivity NGS Fragment Analysis Kit (Advanced Analytical Technologies Inc.). Samples were then pooled, followed by emulsion PCR on the Ion OneTouch 2 system with Ion PI Template OT2 200 Kit v3 (Thermo Fisher Scientific) and enrichment using the Ion OneTouch ES (Thermo Fisher Scientific). Samples were loaded on an Ion PI chip Kit v3 and sequenced in the Ion Proton System using the Ion PI^™^ Sequencing 200 Kit v3 (Thermo Fisher Scientific).

### Bioinformatic analysis of RNA sequencing data

Acquired reads from RNA Seq were analyzed using the hg19 AmpliSeq RNA plugin in the Torrent Suite Server with default settings. This analysis tool produces tables with raw read counts as well as normalized expression values for all genes in the Ion AmpliSeq Human Transcriptome panel. For each experiment, the RPKM values were normalized across samples using the moose2 software (https://github.com/grabherr/moose2). Differential expression was estimated based on Laplace/Normal distributions calculated from replicate samples, computing individual significance values as

$$p_{i}^{j}{}^{\left(+\right)}=2(1-cdf(c\;\text{ln}(r_{i})-ln(r_{j})))$$

$$p_{i}^{j}{}^{\left(-\right)}=2(1-cdf(c\;\text{ln}(r_{j})-ln(r_{i})))$$

with *r*_*i*_ and *r*_*j*_ being the RPKM values, for up-and down-regulation respectively for all given pairwise comparisons *i* and *j*, applying a correction term *c* based on a normal distribution of read counts. The expectations $E_{k}^{l}{}^{\left(+\right)}$ and $E_{k}^{l}{}^{\left(-\right)}$ for comparing all replicates from condition *k* with *l* testing for up- or down-regulation in time point *k* were estimated as

$$E_{k}^{l}{}^{\left(+,-\right)}=n\prod_{i}^{j}p_{i}^{j}{}^{\left(+,-\right)}$$

for all $p_{i}^{j}>0$ and n being the number of $p_{i}^{j}$ values, and Bonferroni-corrected to account for multiple testing. For reporting significance, a threshold of E < 0.00001 was applied.

Genes with significant change in the level of transcription were also subject to GO Terms analysis (http://geneontology.org/page/go-enrichment-analysis) to identify biological or molecular functions as well as protein class. We used the GO slim option with Bonferroni correction applied for multiple testing (*P* < 0.05).

### Quantitative polymerase chain reaction (qPCR)

For qPCR experiments, RNA was extracted as above and cDNA was synthesized from 1μg RNA using the RevertAid H Minus First Strand cDNA Synthesis Kit (Thermo Fisher Scientific) following the manufacturer’s instructions. qPCR was performed on the genes, *il-8*, *cxcl1-3*, *ccl2* and *ccl20* with *gapdh* used as an endogenous control. The Maxima SYBR Green/ROX qPCR Master Mix (Thermo Fisher Scientific) was used in the qPCR reactions with 250 nM of each primer and 10 ng template. Cycling conditions included an initial polymerase heat activation step (10 min, 95°C), and 40 cycles of 15 sec denaturation (95°C), 30 sec annealing (60°C) and 30 sec extension (72°C). The change in gene transcription was calculated using the ΔΔCt method.

### Activator protein 1 (AP-1) reporter plasmid luciferase assay

AP-1 is a transcription factor involved in inducing the transcription of inflammatory genes [28,29]. It is activated by MAPKs and could work in conjunction with NFκB to activate gene transcription [28,29]. To detect AP-1 activation in response to *Giardia* ESPs, Caco-2 cells were transfected with a plasmid pGL4.44[luc2P/AP1 RE/Hygro] expressing the luciferase reporter gene *luc2P* (*Photinus pyralis*) under the control of a promoter that has the transcription response element of AP-1. The pGL4.75 Vector (encoding *Renilla* luciferase) was used as a normalisation control and was co-transfected into Caco-2 cells with the pGL4.44 plasmid. Both plasmids were purchased from Promega (WI, USA). Transfections were performed in a 24-wells plate format according to the supplier’s protocol, using 1.1μg of plasmid DNA/10^5^ cells and the FuGENE HD reagent (Promega). In the beginning of experiment, both parasite and Caco-2 cells were washed and processed as described earlier and the wells were replenished with 500 μl DMEM (no phenol red as it interferes with luminescence reading). Trophozoites were re-suspended in DMEM (no phenol red) and 10^6^ trophozoites were added into the Transwell insert in a total volume of 100 μl and incubated for 2h or 6h (37°C, 10% CO_2_). Three wells were used for each time point and the controls. At the end of each incubation, the inserts were removed and the cells were washed (3x) with 200 μl PBS at RT, and lyzed with an equal amount of the Dual-Glo Luciferase Assay reagent. A 100 μl was dispensed into the well of 96-well plate in triplicates and luminescence was read from both plasmids following the instructions of Dual-Glo Luciferase Assay reagent and using an Infinite M200 Pro Tecan machine (Tecan Group Ltd., Männedorf, Switzerland). Cells with no plasmid (background fluorescence), cells with plasmid alone (negative control), and cells incubated with Phorbol 12-myristate 13-acetate (PMA) (positive control) served as controls. Three biological replicates were performed in this experiment.

### Western blot analyses

To correlate RNA Seq results with those at protein level, we studied the effects of *Giardia* ESPs on differentiated Caco-2 cells using Western blotting. We tested for the activation/inhibition of mitogen activated protein kinases (MAPKs) and the nuclear translocation of nuclear factor kappa-light-chain-enhancer of activated B cells (NF-κB), both of which are involved in inflammatory gene transcription [30,31]. Briefly, the insert experiment was repeated as described earlier with the inclusion of three washes with warm PBS (37°C) at the end of each incubation. In the last wash, cells were scraped off and pelleted by centrifugation (930 x*g*, 10 min, 4°C) and the pellet was lyzed using the NE-PER Nuclear and Cytoplasmic Extraction Reagents (Thermo Fisher Scientific) according to supplier’s instructions. Both Halt Protease and Phosphatase Inhibitor Cocktails (Thermo Fisher Scientific) were added to lysis buffers during extractions. Cytoplasmic and nuclear fractions were aliquoted and stored at -80°C until later use. To confirm that *Giardia* ESPs can modulate/inhibit immune signaling in IECs, we repeated the experiment above but IECs were previously treated with lipopolysaccharide (LPS, Sigma-Aldrich) (10 μg/ml) or tumour necrosis factor-alpha (TNF-α, Sigma-Aldrich) (100 ng/ml) for 2h prior adding trophozoites into the inserts. Trophozoites in inserts and IECs, in the presence of inflammatory signal, were left to interact for 2h and 6h, upon which IECs were processed as above for protein collection. IECs treated with LPS and TNF-α were tested for MAPKs activation/inhibition and the nuclear translocation of NF-κB.

Protein samples collected as described above were used for SDS-PAGE and Western blots. Briefly, 30 μg of total proteins were electrophoresed on AnykD-stain-free gels (Bio-Rad) (100 v, 4°C) and transferred onto a PVDF membrane (100 v, 90 min, 4°C). Membranes were washed with Tris-Buffered Saline with 0.1% Tween-20 (TBST) and blocked with 5% non-fat dry milk in TBST for 1h at RT, followed by incubation with primary antibodies overnight (4°C). Blots were then washed with TBST (3x) and incubated for 1h at RT with a secondary anti-mouse HRP conjugated or anti-rabbit HRP conjugated antibodies (1:5000 dilution, Cell Signaling Technology, MA, USA). Blots were developed using the Clarity ECL Western Blotting Substrate (Bio-Rad). For re-probing, blots were stripped using a mild (200 mM glycine, 0.1% w/v SDS, 1% Tween 20, pH 2.2) or harsh stripping buffer (2% SDS, 62.5 mM Tris, pH 6.8, and 114.4 mM beta-mercaptoethanol) and probed for the loading control, which is either an anti-human GAPDH (cat no. MAB5718, R&D Systems, Inc., MN, USA), anti α-tubulin antibody (Cell Signaling Technology) or TATA box binding protein (TBP) for nuclear samples (MA5-14739, Thermo Fisher Scientific). To detect the activation of MAPKs (ERK1/2, P38 and JNK), the following antibodies were used against the phosphorylated and non-phosphorylated forms of kinases: phospho-p44/42 MAPK (Erk1/2) (cat no. 8544) and p44/42 MAPK (Erk1/2) (cat no. 4348), Phospho-p38 MAPK (cat no. 4511) and p38 MAPK (cat no. 8690), Phospho-SAPK/JNK (cat no. 4671) and SAPK/JNK (cat no. 9258), and NF-κB1 p105/p50 (cat no. 13586). Antibodies were purchased from Cell Signaling Technology and used at the dilution recommended by the supplier.

### Immunofluorescence of labelled ESPs and confocal microscopy

For ESPs labelling with Alexa Fluor 488, proteins collected from axenic culture were washed (3x) with cold PBS during sample concentration to remove free amino acids and antioxidants that might interfere with protein labelling (recommended in the kit). ESPs (45μg) were incubated with 11.3 nmol/μl of Alexa Fluor-488 and excess dye was removed by gel filtration following the instructions in the Alexa Fluor 488 Microscale Protein Labelling Kit (Thermo Fisher Scientific).

To visualise ESPs interaction with host cells, chamber slides were washed and replenished with fresh DMEM as above, followed by adding labelled ESPs to the wells at 1 or 5 μg/ml and incubation for 2 h or 6 h (37°C, 10% CO_2_). The amounts of labelled proteins were quantified using a NanoDrop 1000 Spectrometer (Thermo Fisher Scientific). Caco-2 cells incubated in DMEM only (no Labelled ESPs added) served as a control. Slides were washed, fixed (4% paraformaldehyde in PBS) and mounted (Vectashield anti-fade reagent with DAPI, Vector Laboratories, CA, USA), excluding the blocking step. The fluorescent label on IECs was visualised using a Zeiss LSM700 confocal microscope (Zeiss, Oberkochen, Germany) at the BioVis Core Facility, SciLifeLab, Uppsala, Sweden.

### Statistical analyses

The results from the cytokine measurements and differential gene expression for qPCR results were analysed using a one-way analysis of variance (ANOVA) to assess overall significance among the tested groups at *α* < 0.05. When significant, the values from the replicates were processed through the Bonferroni-Holm’s and significance was marked at *P* < 0.05.

## Results

### The secretome of *Giardia* trophozoites in the absence and presence of host cells

**Fig 2:**
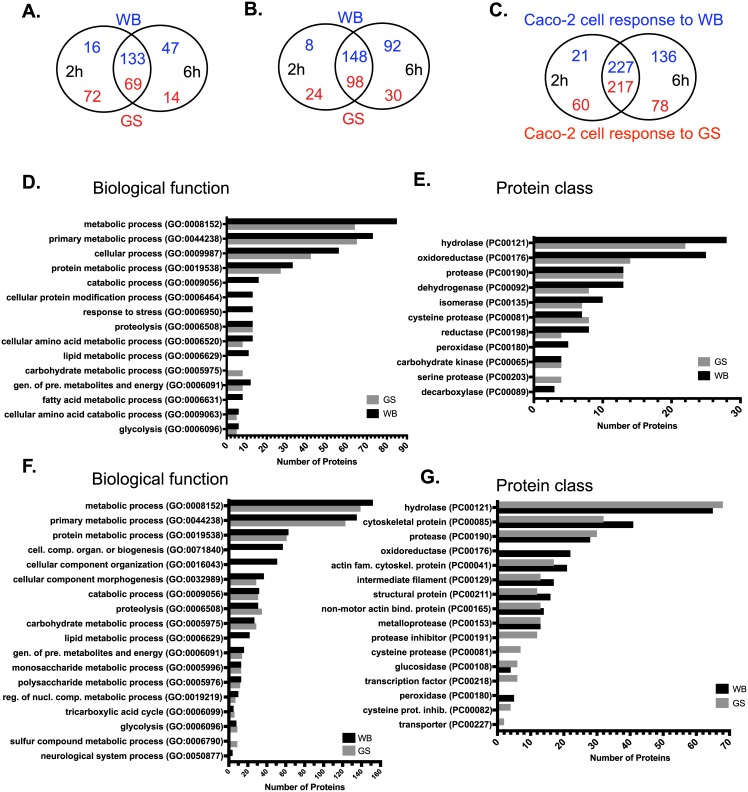
Secretomes of *Giardia* trophozoites and differentiated Caco-2 cells during interactions. (A) Number of secreted WB and GS proteins in axenic culture. (B) Number of secreted WB and GS proteins during host cell interactions. (C) Number of secreted Caco-2 cell proteins during interaction with *Giardia* WB and GS trophozoites. (D) and (E) GO term analyses of WB and GS secretomes during interaction with host cells. (F) and (G) GO term analyses of secreted Caco-2 cell proteins during interactions with *Giardia* WB and GS trophozoites.

In order to identify proteins in the *Giardia* secretome, we collected ESPs from *Giardia* trophozoites grown axenically or in the presence of IECs (Fig 1A). The secretomes of axenic WB (assemblage A) and GS (assemblage B) *Giardia* trophozoites were analyzed in a modified serum-free medium (see Materials and methods) that could support the full viability of trophozoites for 2h and estimated viabilities of 98.74% ± 1.03 (WB, n = 5) and 98.32% ± 0.81 (GS, n = 5) under 6h of incubation. In total, 196 proteins were identified in the secretome of the WB isolate with 149 proteins detected in 2h culture and 180 in 6h culture; 133 proteins overlapped in both time points (Fig 2A). For the GS isolate, 155 proteins were identified including 141 proteins from the 2h culture and 83 proteins from 6h culture; 69 proteins overlapped in both time points (Fig 2A). The reproducibility of the secretome profiles was conserved in the three biological replicates. Full lists of identified proteins, including tryptic peptides, score, coverage and number of peptide spectrum matches (PSM) are presented in S1 Table (WB) and S2 Table (GS). The secretomes of both isolates were also compared leading to the identification of 122 common secreted proteins (S3 Table).

We also collected and analyzed the medium from *Giardia* trophozoites incubated with differentiated Caco-2 cells (Fig 1A). In total, 248 WB proteins were identified in the co-culture medium, 156 at 2h, 240 at 6h and 148 in both time points (Fig 2B, S4 Table). For the GS isolate, 152 proteins in total were identified, 122 at 2h, 128 at 6h and 98 at both time points (Fig 2B, S5 Table). The top 50 proteins, based on their peptide score, are listed in Table 1. These proteins overlap with those in axenic culture (S1 and S2 Tables). A comparison was performed between the secretomes of WB trophozoites incubated with (S4 Table) and without IECs (S1 Table) through which 161 common proteins and 87 interaction-specific proteins were identified (Table 2). Similarly, an analysis of the secreted proteins from GS isolate with (S5 Table) and without IECs (S2 Table) identified 111 common proteins and 41 proteins specific to the interaction (Table 2). Amongst the proteins specific to interactions, 13 proteins overlap between the two isolates (denoted in Table 2) whereas 74 WB trophozoite proteins and 28 GS trophozoite proteins are exclusive to the interaction process and represent an isolate-specific response to IECs. This overall shows that most of the secreted *Giardia* proteins during interactions with IECs are conserved between the WB and GS isolates but there are assemblage-specific differences.

**Table 1:** Top 50 identified proteins of *Giardia intestinalis* isolates, WB and GS, secreted into the medium of interaction with intestinal epithelial cells *in vitro*. The proteins are ranked based on their peptide score. Note that all listed proteins were also detected in axenic cultures.

ORF (WB)	Protein name	Score	ORF (GS)	Protein name	Score
**GL50803_112103**	Arginine deiminase	1547,4	**GL50581_668**	Ornithine carbamoyltransferase	1098,26
**GL50803_10311**	Ornithine carbamoyltransferase	794,21	**GL50581_1575**	Arginine deiminase	782,58
**GL50803_14019**	Cathepsin B precursor	477,17	**GL50581_1499**	Alanyl dipeptidyl peptidase	779,52
**GL50803_11043**	Fructose-bisphosphate aldolase	443,25	**GL50581_4371**	Enolase	719,61
**GL50803_8826**	Glucokinase	402,97	**GL50581_3654**	Phosphomannomutase-2	625,1
**GL50803_17476**	CXC-rich protein	400,75	**GL50581_4115**	Fructose-bisphosphate aldolase	527,43
**GL50803_10330**	Tenascin precursor	374,9	**GL50581_3472**	Inosine-uridine nucleoside N-ribohydrolase	463,7
**GL50803_16160**	Cathepsin B precursor	371,65	**GL50581_1886**	Inositol-3-phosphate synthase	427,19
**GL50803_8822**	2,3-bisphosphoglycerate-independent phosphoglycerate mutase	332,29	**GL50581_78**	Cathepsin B precursor	395,87
**GL50803_11118**	Enolase	329,33	**GL50581_945**	2,3-bisphosphoglycerate-independent phosphoglycerate mutase	333,4
**GL50803_15574**	Alanyl dipeptidyl peptidase	327,22	**GL50581_661**	Hypothetical protein	277,38
**GL50803_3042**	Hybrid cluster protein lateral transfer candidate	292,29	**GL50581_2060**	Alanyl dipeptidyl peptidase	252,55
**GL50803_104250**	hypothetical protein	243,21	**GL50581_2343**	Carbamate kinase	241,43
**GL50803_16779**	Cathepsin B precursor	220,7	**GL50581_943**	Glucokinase	222,28
**GL50803_17254**	Phosphomannomutase-2	208,7	**GL50581_3133**	Hypothetical protein	180,7
**GL50803_480**	Translation initiation inhibitor	188,68	**GL50581_2946**	Cathepsin B precursor	166,48
**GL50803_17327**	Xaa-Pro dipeptidase	179,82	**GL50581_4017**	Translation initiation inhibitor	157,82
**GL50803_15409**	Kinase, NEK	179,42	**GL50581_770**	Transketolase	156,3
**GL50803_91056**	Aspartate aminotransferase, cytoplasmic	178,13	**GL50581_438**	Cathepsin B precursor	128,34
**GL50803_10358**	A-type flavoprotein lateral transfer candidate	173,86	**GL50581_2728**	Alcohol dehydrogenase 3 lateral transfer candidate	123,66
**GL50803_3910**	hypothetical protein	172,18	**GL50581_2834**	Dynein light chain	115,96
**GL50803_17153**	Alpha-11 giardin	169,28	**GL50581_1568**	Acetyl-CoA synthetase	115,07
**GL50803_10623**	Phosphoenolpyruvate carboxykinase	165,9	**GL50581_2473**	Alpha-11 giardin	112,02
**GL50803_13272**	hypothetical protein	143,1	**GL50581_1523**	UPL-1	110,31
**GL50803_88765**	Cytosolic HSP70	121,97	**GL50581_1192**	Pyrophosphate-fructose 6-phosphate 1-phosphotransferase alpha subunit	109,71
**GL50803_27925**	Protein 21.1	119,86	**GL50581_3386**	Protein disulfide isomerase PDI5	108,36
**GL50803_17579**	Inositol-3-phosphate synthase	118,4	**GL50581_2893**	Aspartate aminotransferase, cytoplasmic	103,66
**GL50803_17570**	Elongation factor 2	117,26	**GL50581_4057**	Tenascin precursor	101,34
**GL50803_93938**	Triosephosphate isomerase, cytosolic	114,07	**GL50581_413**	Elongation factor 1-alpha	99,37
**GL50803_17277**	Phospholipase B	112,21	**GL50581_4499**	Hypothetical protein	96,87
**GL50803_11599**	Lysophosphatidic acid phosphatase	105,32	**GL50581_881**	Hypothetical protein	88,04
**GL50803_93548**	Phospholipase B	101,25	**GL50581_1765**	Elongation factor 2	84,44
**GL50803_7260**	Aldose reductase	98,33	**GL50581_4335**	Ribokinase	83,39
**GL50803_12150**	Alanine aminotransferase, putative	97,57	**GL50581_2786**	VSP	83,36
**GL50803_1875**	hypothetical protein	97,36	**GL50581_1032**	Protein 21.1	80,38
**GL50803_16468**	Cathepsin B precursor	95,85	**GL50581_2036**	Cathepsin B precursor	79,55
**GL50803_9848**	Dynein light chain	94,85	**GL50581_521**	Glyceraldehyde 3-phosphate dehydrogenase	78,92
**GL50803_4039**	Leucine-rich repeat protein	91,16	**GL50581_4262**	Leucine-rich repeat protein	78,4
**GL50803_15317**	High cysteine membrane protein Group 1	90,72	**GL50581_1019**	Peptidyl-prolyl cis-trans isomerase B precursor	77,03
**GL50803_5845**	Ribosomal protein S8	87,79	**GL50581_821**	Leucine-rich repeat protein 1 virus receptor protein	76,69
**GL50803_17060**	Protein 21.1	87	**GL50581_31**	Neurogenic locus Notch protein precursor	75,81
**GL50803_11354**	hypothetical protein	82,67	**GL50581_3442**	Malate dehydrogenase	71,15
**GL50803_7796**	Alpha-2 giardin	81,99	**GL50581_1672**	Alpha-2 giardin	69,04
**GL50803_14670**	PDI3—Protein disulfide isomerase	81,26	**GL50581_380**	Phospholipase B	66,96
**GL50803_6148**	Alanyl dipeptidyl peptidase	80,76	**GL50581_3909**	Hypothetical protein	64,53
**GL50803_3313**	Serine-pyruvate aminotransferase	80,61	**GL50581_1587**	Giardia trophozoite antigen GTA-1	63,59
**GL50803_16376**	ATP-dependent RNA helicase p47, putative	80,37	**GL50581_1047**	Pyruvate-flavodoxin oxidoreductase	62,64
**GL50803_15297**	Ribokinase	75,65	**GL50581_2467**	20S proteasome alpha subunit 4	60,53

**Table 2:** Proteins specifically released by *Giardia intestinalis* isolates, WB and GS, during interaction with differentiated Caco-2 cells.

Protein ID	Protein name	WB		Protein ID	Protein name	GS	
		2h	6h			2h	6h
GL50803_16839	Kinase, NEK-frag			GL50581_4493	Hypothetical protein		
GL50803_16824	Kinase, NEK			GL50581_403	Cyst wall protein 1		
GL50803_9355	Hypothetical protein			GL50581_3511	Ribosomal protein L34		
GL50803_8942	Hypothetical protein			GL50581_3236	CXC-rich protein		
GL50803_8407	Aminoacyl-histidine dipeptidase			GL50581_1034	Protein 21.1		
GL50803_8245	Glucosamine-6-phosphate deaminase			GL50581_3117	Kinase, NEK		
GL50803_8064*	PDI5—Protein disulfide isomerase			GL50581_2562	Ribosomal protein S2		
GL50803_7195	Glutamate synthase			GL50581_2221	Kinesin like protein		
GL50803_7082*	Ribosomal protein S21			GL50581_2131	Phosphoenolpyruvate carboxykinase		
GL50803_6535	Hypothetical protein			GL50581_1670	Alpha-6 giardin		
GL50803_4912	Kinase, NEK			GL50581_1600	Ribosomal protein S7		
GL50803_4156	HIT family protein			GL50581_1186	Peptidyl-prolyl cis-trans isomerase B precursor		
GL50803_4059	5-methylthioadenosine nucleosidase, S-adenosylhomocysteine nucleosidase			GL50581_823	Hypothetical protein		
GL50803_3593	Alcohol dehydrogenase lateral transfer candidate			GL50581_583	Kinesin-3		
GL50803_22850	Kinase, CMGC MAPK			GL50581_4527	Kinase, NEK		
GL50803_17231	Kinase, NEK			GL50581_4146	Alpha-14 giardin		
GL50803_17060	Protein 21.1			GL50581_3698	Hypothetical protein		
GL50803_16180	Protein phosphatase methylesterase-1			GL50581_3607	Extracellular nuclease, putative		
GL50803_15411*	Kinase, NEK-frag			GL50581_3593	FKBP-type peptidyl-prolyl cis-trans isomerase		
GL50803_14841	Phosphoglycolate phosphatase			GL50581_3370	Protein disulfide isomerase PDI5		
GL50803_14497	20S proteasome alpha subunit 3			GL50581_203	4-methyl-5-thiazole monophosphate biosynthesis enzyme		
GL50803_14434	Protein 21.1			GL50581_1997	Hypothetical protein		
GL50803_137747	Hypothetical protein			GL50581_186	Protein 21.1		
GL50803_13561	Translation elongation factor			GL50581_128	Phospholipase B		
GL50803_13350	Alcohol dehydrogenase lateral transfer candidate			GL50581_897	Hypothetical protein		
GL50803_114609	Pyruvate-flavodoxin oxidoreductase			GL50581_884	Hypothetical protein		
GL50803_113319	High cysteine membrane protein TMK-like			GL50581_73	Hypothetical protein		
GL50803_101339*	FKBP-type peptidyl-prolyl cis-trans isomerase			GL50581_493	Hypothetical protein		
GL50803_9899	Hypothetical protein			GL50581_4466	Nucleolar protein NOP5		
GL50803_9620	High cysteine membrane protein Group 2			GL50581_3346	Rab11		
GL50803_95593	Kinase, NEK			GL50581_3318	Hypothetical protein		
GL50803_9413	PDI2—Protein disulfide isomerase			GL50581_3249	Ribosomal protein S21		
GL50803_93358	Alcohol dehydrogenase			GL50581_3195	VSP		
GL50803_91504	Hypothetical protein			GL50581_2900	Protein 21.1		
GL50803_9088*	4-methyl-5-thiazole monophosphate biosynthesis enzyme			GL50581_2665	Kinase, NEK		
GL50803_9030	Protein 21.1			GL50581_2356	Spindle pole protein, putative		
GL50803_8742*	Extracellular nuclease, putative			GL50581_210	Hypothetical protein		
GL50803_8726	Actin related protein			GL50581_1952	Hypothetical protein		
GL50803_7843*	Hypothetical protein			GL50581_183	Kinase, NEK		
GL50803_7797	Alpha-5 giardin			GL50581_1635	Kinase, NEK		
GL50803_7268	Protein 21.1			GL50581_1034	Protein 21.1		
GL50803_7110	Ubiquitin						
GL50803_7031*	Spindle pole protein, putative						
GL50803_7030	Prefoldin subunit 3, putative						
GL50803_6289	FixW protein, putative						
GL50803_6242	Translationally controlled tumor protein-like protein						
GL50803_5942	Polyadenylate-binding protein, putative						
GL50803_5346	Kinase, NEK						
GL50803_5188	Protein 21.1						
GL50803_4571	Hypothetical protein						
GL50803_4204	MutT/nudix family protein						
GL50803_3861	Alcohol dehydrogenase 3 lateral transfer candidate						
GL50803_3822	Dipeptidyl-peptidase III						
GL50803_34050	Hypothetical protein						
GL50803_33769	NADH oxidase lateral transfer candidate						
GL50803_29490	Copine I						
GL50803_29307	UTP-glucose-1-phosphate uridylyltransferase						
GL50803_24947	Hypothetical protein (VPS46b)						
GL50803_23004	Hypothetical protein						
GL50803_21942	NADP-specific glutamate dehydrogenase						
GL50803_21505	Protein 21.1						
GL50803_2101	Cytidine deaminase						
GL50803_17563	Kinase, CMGC MAPK						
GL50803_16817	Transglutaminase/protease, putative						
GL50803_16717*	Hypothetical protein						
GL50803_16532	Protein 21.1						
GL50803_16353	Hypothetical protein						
GL50803_16326	Protein 21.1						
GL50803_16069	Phosphoacetylglucosamine mutase						
GL50803_15472	Hypothetical protein (VPS46a)						
GL50803_15097*	Alpha-14 giardin						
GL50803_14737	Hypothetical protein						
GL50803_14521	Peroxiredoxin 1						
GL50803_137698	Sec13 (Sec13)						
GL50803_13766*	Protein 21.1						
GL50803_135885	Hypothetical protein						
GL50803_13127	Proteasome subunit beta type 1						
GL50803_114119	Alpha-7.2 giardin						
GL50803_113970	Hypothetical protein						
GL50803_11359	Ribosomal protein S4						
GL50803_113531	High cysteine membrane protein EGF-like						
GL50803_112846*	Kinesin-3						
GL50803_11165*	Protein 21.1						
GL50803_104685	Caltractin						
GL50803_10450	FKBP-type peptidyl-prolyl cis-trans isomerase						
GL50803_10315	Qb-SNARE 5						
GL50803_102813	Protein 21.1						

* Common proteins released by WB and GS isolates in response to host cells

Secreted *Giardia* proteins in co-culture were analyzed for the enrichment of certain biological functions and for protein class. For the GS isolate, since the current online algorithms do not support GO analysis, orthologous genes in WB were used to perform the quest. The results are presented in Fig 2D and 2E. The largest number of detected proteins have functions associated with metabolism (85 proteins in WB and 64 in GS) with different subgroups in the child linages associated with protein metabolism, amino acid catabolic and metabolic processes, glycolysis, monosaccharide metabolic process and the generation of precursor metabolites and energy (Fig 2D). These metabolic groups were also seen in the secretome of axenic cultures (S1 Fig). Other metabolic functions such as the hexose monophosphate shunt and arginine metabolism could also be identified when secreted proteins were manually delineated into different metabolic pathways (S1 Fig). Furthermore, a nuclease with endonuclease I activity (Table 1), possibly involved in nucleic acids salvaging, and proteins involved in pyrimidine salvage (cytidine deiminase and uridine phosphorylase (UPL-1)) as well as in lipid metabolism were identified in secretome of co-culture (S1 Fig). The lipid metabolic proteins included enzymes involved in phospholipids (PLs) remodelling and salvaging fatty acids (phospholipase B, PLB), producing monoacylglycerol (lysophospholipid phosphatase) and generating inositol-3 phosphate (I3P, I3P synthase), the precursor for all inositol containing compounds including PLs [32]. These result show that the major part of *Giardia* secretome is involved in different metabolic functions.

The majority of proteins in the two isolates metabolo-secretome fell under four major categories: the hydrolase, dehydrogenase, oxidoreductase and protease groups. The oxidoreductase group contained 25 proteins in WB and 14 proteins in GS (Fig 2E). Compared with axenic culture (14 proteins) (S2A Fig), the WB isolate released 11 additional proteins with oxidoreductase activity including peroxidases when incubated with IECs (e.g. PFOR, NADH oxidase lateral transfer candidate, peroxiredoxin 1, FixW protein alcohol dehydrogenases (ALDs), glutamate dehydrogenase and glutamate synthase and a HP GL50803_9355, Table 2). This suggests a response to counteract IECs oxidative defences such as reactive oxygen species. It has been suggested that oxidative stress provides the trigger to initiate encystation [33] and proteins involved in cyst wall synthesis (e.g. glucosamine 6-phosphate deaminase and N-acetylglucosamine 6-phosphate mutase) were released by WB isolate interacting with IECs, corroborating this notion. For GS, on the other hand, peroxidases and antioxidant proteins were already secreted by trophozoites without exposure to IECs (S2 Table) and thus the results above show that the two isolates exhibit different anti-oxidative responses during interaction with IECs.

The protease group contained 13 secreted proteins in both isolates (Fig 2E) and harboured the cysteine- (cathepsins B and L), serine- (Alanyl dipeptidyl peptidases) and metallo-type (Aminoacyl-histidine dipeptidases and Xaa-Pro dipeptidases) of proteases. For WB isolate, two secreted metalloproteases (GL50803_8407 and GL50803_3822) were released in response to IECs whereas the rest of proteases were the same as those in axenic culture. For GS isolate, the same proteases were detected in both co-culture and axenic culture with two serine proteases (GL50581_3181 and GL50581_2704) being non-orthologous to any WB isolate proteins.

Many of the proteins detected in our analysis have been previously reported as immunoreactive proteins during human giardiasis (S6 Table) [21]. Amongst these proteins, the variable surface proteins (VSPs) are immunodominant during infection [2] and together with the high cysteine membrane proteins (HCMPs) they occupied a large portion of the WB isolate secretome (23 and 12, respectively, S1 Table). In the GS isolate secretome, lower numbers of VSPs and HCMPs were detected (4 and 5, respectively, S2 Table), more likely due to the fragmented genome used in protein identification. VSPs and HCMPs are cysteine-rich proteins and those detected in our analysis have an epidermal growth factor (EGF)-like domain in common (InterPro:IPR000742, 39 in WB and 10 in GS) which is also found in other secreted cysteine-rich proteins (e.g. tenascins, high cysteine proteins (HCPs) and neurogenic locus Notch protein precursor, S1 and S2 Tables). EGF-like domains are often present in secreted protein [34] and thus, this promoted us to look for proteins with secretion signal peptides (SSPs). A genome-wide screen in the *Giardia* database showed that 1361 proteins of the WB isolate have SSPs, amongst which 82 proteins were detected in our co-culture secretome analysis (~29%, S7 Table) with 41 of those being N-glycosylated [35] (S7 Table). For the GS isolate, 422 proteins in *Giardia* DB contain SSPs, 44 were detected in our analysis (~29%, S7 Table). The majority of proteins with SSPs (S7 Table) contained either an EGF-like domain (38 proteins for WB and 16 for GS) or a peptidase C1A domain (8 proteins for WB and 8 proteins for GS). The detection of a relatively small number of proteins with SSPs in *Giardia* secretomes was not surprising as this has been previously reported in other protozoan parasites like *Trypanosoma*. *brucei* (~20%) and *Leishmania donovani* (~9%) [36,37], suggesting the presence of alternative secretory pathways. Many protozoan parasites produce extracellular vesicles (MVs and exosomes), which can release proteins without SPPs [38]. MVs have recently been shown to be released from *Giardia* trophozoites [26] and we could detect two types of vesicles (100–250 nm and 100 nm, S2B Fig) in the supernatants of axenic trophozoite culture. These vesicles might explain the release of proteins without SSPs but further experiments are needed to show how these vesicles are formed and what proteins they contain.

### The secretome of differentiated Caco-2 cells during parasite interactions

The secretome of differentiated Caco-2 cells incubated with WB trophozoites was analyzed for proteins released in co-culture. Overall, 384 Caco-2 cell proteins were identified in the medium of interaction with 21 proteins secreted at 2h, 136 at 6h and 227 at both time points (Fig 2C). The identified proteins, together with the number of peptides, score, coverage and PSMs are presented in S4 Table. A comparison between the secretomes of Caco-2 cells incubated alone (i.e. control, S8 Table) and with WB trophozoites (S4 Table) identified 308 overlapping proteins (S9 Table) and 76 proteins released in response to WB trophozoites (Table 3). The secretome of Caco-2 cells incubated with GS trophozoites was also analyzed for the proteins released by host cells in response to interaction. In total, 355 proteins were secreted in co-culture, 60 proteins were secreted at 2h, 78 at 6h and 217 at both time points (Fig 2C). The proteins identified, together with the number of peptides, score, coverage and PSMs are presented in S5 Table. The top 50 proteins, based on their peptide scores, are presented in Table 4. A comparison between the secretomes of co-culture (S5 Table) and control (S8 Table) identified 310 common proteins (S10 Table) and 45 proteins that were specifically released in response to GS trophozoites (Table 3). Amongst the proteins specific to interaction (45 proteins) 31 proteins overlapped in the response to both isolates.

**Table 3:** Proteins secreted by differentiated Caco-2 cells specific to interaction with *Giardia intestinalis* isolates, WB and GS.

Protein ID	Protein name	WB		Protein ID	Protein name	GS	
		2h	6h			2h	6h
Q9UHB6	LIM domain and actin-binding protein 1			Q9Y2V2	Calcium-regulated heat stable protein 1		
Q8NF91*	Nesprin-1			Q9UKM9	RNA-binding protein Raly		
Q6XZF7	Dynamin-binding protein			Q9P2E9	Ribosome-binding protein 1		
P62081*	40S ribosomal protein S7			Q9NSK0	Kinesin light chain 4		
P35637	RNA-binding protein FUS			Q8NF91	Nesprin-1		
P35579	Myosin-9			Q15836	Vesicle-associated membrane protein 3		
P26641	Elongation factor 1-gamma			Q15493	Regucalcin		
P11234*	Ras-related protein Ral-B			Q13148	TAR DNA-binding protein 43		
O00151*	PDZ and LIM domain protein 1			Q09666	Neuroblast differentiation-associated protein AHNAK		
Q9Y5Y6	Suppressor of tumorigenicity 14 protein			P62081	40S ribosomal protein S7		
Q9ULC5*	Long-chain-fatty-acid—CoA ligase 5			P46108	Adapter molecule crk		
Q9ULA0	Aspartyl aminopeptidase			P42167	Lamina-associated polypeptide 2, isoforms beta/gamma		
Q9P2E9*	Ribosome-binding protein 1			P40925	Malate dehydrogenase, cytoplasmic		
Q9BS26	Endoplasmic reticulum resident protein 44			P38159	RNA-binding motif protein, X chromosome		
Q9BQE3	Tubulin alpha-1C chain			P31939	Bifunctional purine biosynthesis protein PURH		
Q99729	Heterogeneous nuclear ribonucleoprotein A/B			P30085	UMP-CMP kinase		
Q7Z406	Myosin-14			P21333	Filamin-A		
Q15149	Plectin			P16083	Ribosyldihydronicotinamide dehydrogenase [quinone]		
Q13263	Transcription intermediary factor 1-beta			P14174	Macrophage migration inhibitory factor		
Q13148*	TAR DNA-binding protein 43			P11234	Ras-related protein Ral-B		
P55795	Heterogeneous nuclear ribonucleoprotein H2			P09874	Poly [ADP-ribose] polymerase 1		
P52597*	Heterogeneous nuclear ribonucleoprotein F			P07437	Tubulin beta chain		
P43487	Ran-specific GTPase-activating protein			P01024	Complement C3		
P42167	Lamina-associated polypeptide 2, isoforms beta/gamma			O60610	Protein diaphanous homolog 1		
P38159*	RNA-binding motif protein, X chromosome			Q9H6S3	Epidermal growth factor receptor kinase substrate 8-like protein 2		
P31939*	Bifunctional purine biosynthesis protein PURH			Q14980	Nuclear mitotic apparatus protein 1		
P29692	Elongation factor 1-delta			P52597	Heterogeneous nuclear ribonucleoprotein F		
P22626	Heterogeneous nuclear ribonucleoproteins A2/B1			P13797	Plastin-3		
P21333*	Filamin-A			O60437	Periplakin		
P20700*	Lamin-B1			O00151	PDZ and LIM domain protein 1		
P14174*	Macrophage migration inhibitory factor			Q9ULC5	Long-chain-fatty-acid—CoA ligase 5		
P0CW22	40S ribosomal protein S17-like			Q9HCB6	Spondin-1		
P09327	Villin-1			Q92896	Golgi apparatus protein 1		
P07437*	Tubulin beta chain			Q8NCL4	Polypeptide N-acetylgalactosaminyltransferase 6		
O60610*	Protein diaphanous homolog 1			Q14031	Collagen alpha-6(IV) chain		
O60437*	Periplakin			P61758	Prefoldin subunit 3		
O43169	Cytochrome b5 type B			P22304	Iduronate 2-sulfatase		
Q9Y2V2*	Calcium-regulated heat stable protein 1			P20700	Lamin-B1		
Q9UPA5	Protein bassoon			P15291	Beta-1,4-galactosyltransferase 1		
Q9UKM9*	RNA-binding protein Raly			P13010	X-ray repair cross-complementing protein 5		
Q9NZ53	Podocalyxin-like protein 2			P02545	Prelamin-A/C		
Q9NX62	Inositol monophosphatase 3			O95777	U6 snRNA-associated Sm-like protein LSm8		
Q9NR09	Baculoviral IAP repeat-containing protein 6			O94985	Calsyntenin-1		
Q9H6S3*	Epidermal growth factor receptor kinase substrate 8-like protein 2			O75503	Ceroid-lipofuscinosis neuronal protein 5		
Q96HE7	ERO1-like protein alpha			A6NIZ1	Ras-related protein Rap-1b-like protein		
Q92896*	Golgi apparatus protein 1						
Q8WWA0	Intelectin-1						
Q8NDV7	Trinucleotide repeat-containing gene 6A protein						
Q8N8Z6	Discoidin, CUB and LCCL domain-containing protein 1						
Q15084	Protein disulfide-isomerase A6						
Q14980*	Nuclear mitotic apparatus protein 1						
Q12792	Twinfilin-1						
Q09666*	Neuroblast differentiation-associated protein AHNAK						
Q02790	Peptidyl-prolyl cis-trans isomerase FKBP4						
P78386	Keratin, type II cuticular Hb5						
P62750	60S ribosomal protein L23a						
P61758*	Prefoldin subunit 3						
P54687	Branched-chain-amino-acid aminotransferase, cytosolic						
P46108*	Adapter molecule crk						
P40925*	Malate dehydrogenase, cytoplasmic						
P30085*	UMP-CMP kinase						
P15291*	Beta-1,4-galactosyltransferase 1						
P13010*	X-ray repair cross-complementing protein 5						
P12956	X-ray repair cross-complementing protein 6						
P07900	Heat shock protein HSP 90-alpha						
P06576	ATP synthase subunit beta, mitochondrial						
P02545*	Prelamin-A/C						
O95785	Protein Wiz						
O95777*	U6 snRNA-associated Sm-like protein LSm8						
O94985*	Calsyntenin-1						
O76009	Keratin, type I cuticular Ha3-I						
O60814	Histone H2B type 1-K						
O43451	Maltase-glucoamylase, intestinal						
O14638	Ectonucleotide pyrophosphatase/phosphodiesterase family member3						
A8MPP1	Putative ATP-dependent RNA helicase DDX11-like protein 8						
A6NCN2	Putative keratin-87 protein						

* Proteins specifically secreted from differentiated Caco-2 cells in response to both the WB and GS isolates.

**Table 4:** Top 50 identified proteins of secreted by differentiated Caco-2 cells co-incubated with *Giardia intestinalis* isolates, WB and GS *in vitro*. The proteins are ranked based on peptide score. Note that all listed proteins were also detected in Caco-2 cells culture without *Giardia*.

Accession no.	Protein name (response to WB isolate)	Score	Accesion no.	Protein name (response to GS isolate)	Score
**P12277**	Creatine kinase B-type	904,93	**P60174**	Triosephosphate isomerase	1214,66
**P02766**	Transthyretin	895,38	**P12277**	Creatine kinase B-type	1205,28
**P60174**	Triosephosphate isomerase	832,90	**P06733**	Alpha-enolase	1158,98
**P06733**	Alpha-enolase	717,02	**P02766**	Transthyretin	1085,82
**P06744**	Glucose-6-phosphate isomerase	661,49	**P06744**	Glucose-6-phosphate isomerase	891,36
**P04264**	Keratin, type II cytoskeletal 1	458,97	**P15586**	N-acetylglucosamine-6-sulfatase	583,95
**O43895**	Xaa-Pro aminopeptidase 2	442,21	**P09211**	Glutathione S-transferase P	575,74
**P15586**	N-acetylglucosamine-6-sulfatase	430,61	**P01034**	Cystatin-C	568,17
**Q16819**	Meprin A subunit alpha	412,99	**P07148**	Fatty acid-binding protein, liver	526,81
**P08582**	Melanotransferrin	410,69	**Q16819**	Meprin A subunit alpha	502,59
**P35527**	Keratin, type I cytoskeletal 9	402,83	**P14410**	Sucrase-isomaltase, intestinal	437,75
**P09211**	Glutathione S-transferase P	393,19	**P02771**	Alpha-fetoprotein	430,81
**P02771**	Alpha-fetoprotein	355,27	**O43895**	Xaa-Pro aminopeptidase 2	417,56
**P07148**	Fatty acid-binding protein, liver	350,56	**P02765**	Alpha-2-HS-glycoprotein	415,69
**P02765**	Alpha-2-HS-glycoprotein	331,65	**P02753**	Retinol-binding protein 4	402,73
**P01034**	Cystatin-C	320,50	**P07339**	Cathepsin D	364,27
**Q9BYF1**	Angiotensin-converting enzyme 2	314,38	**P08582**	Melanotransferrin	363,96
**P12830**	Cadherin-1	299,38	**Q12907**	Vesicular integral-membrane protein VIP36	362,80
**P07339**	Cathepsin D	298,52	**P01019**	Angiotensinogen	333,26
**P14410**	Sucrase-isomaltase, intestinal	285,68	**P12830**	Cadherin-1	332,52
**P60709**	Actin, cytoplasmic 1	274,83	**P04264**	Keratin, type II cytoskeletal 1	294,79
**Q16651**	Prostasin	251,56	**P06727**	Apolipoprotein A-IV	291,45
**P06727**	Apolipoprotein A-IV	231,08	**P02768**	Serum albumin	266,83
**P07602**	Prosaposin	230,55	**P29401**	Transketolase	265,03
**P02753**	Retinol-binding protein 4	222,88	**P23528**	Cofilin-1	260,02
**Q9HAT2**	Sialate O-acetylesterase	214,79	**Q9HAT2**	Sialate O-acetylesterase	253,00
**Q12907**	Vesicular integral-membrane protein VIP36	213,83	**Q16651**	Prostasin	240,43
**P23141**	Liver carboxylesterase 1	210,24	**P07602**	Prosaposin	236,06
**P05787**	Keratin, type II cytoskeletal 8	200,65	**P62158**	Calmodulin	232,81
**P13645**	Keratin, type I cytoskeletal 10	197,51	**P10253**	Lysosomal alpha-glucosidase	225,21
**P01019**	Angiotensinogen	197,02	**P61769**	Beta-2-microglobulin	211,87
**P62158**	Calmodulin	185,43	**P60709**	Actin, cytoplasmic 1	210,32
**P01009**	Alpha-1-antitrypsin	175,12	**P08758**	Annexin A5	209,71
**P11142**	Heat shock cognate 71 kDa protein	173,21	**P12429**	Annexin A3	208,06
**P12429**	Annexin A3	170,94	**P23141**	Liver carboxylesterase 1	202,09
**P06865**	Beta-hexosaminidase subunit alpha	161,29	**Q02818**	Nucleobindin-1	199,84
**P02647**	Apolipoprotein A-I	158,95	**Q9BYF1**	Angiotensin-converting enzyme 2	186,13
**P02768**	Serum albumin	158,24	**P35527**	Keratin, type I cytoskeletal 9	178,26
**P30041**	Peroxiredoxin-6	157,60	**P09104**	Gamma-enolase	176,25
**P29401**	Transketolase	157,04	**P10599**	Thioredoxin	174,25
**P08758**	Annexin A5	147,23	**P01009**	Alpha-1-antitrypsin	172,46
**P23528**	Cofilin-1	142,84	**P02647**	Apolipoprotein A-I	166,56
**P07711**	Cathepsin L1	141,42	**P35908**	Keratin, type II cytoskeletal 2 epidermal	165,95
**Q02818**	Nucleobindin-1	128,33	**P17174**	Aspartate aminotransferase, cytoplasmic	161,05
**P09104**	Gamma-enolase	124,97	**P06865**	Beta-hexosaminidase subunit alpha	160,17
**P27797**	Calreticulin	123,78	**P07711**	Cathepsin L1	159,94
**P10253**	Lysosomal alpha-glucosidase	118,01	**P13645**	Keratin, type I cytoskeletal 10	157,20
**P10599**	Thioredoxin	117,24	**O75882**	Attractin	146,04

GO enrichment analyses of parasitized Caco-2 cell secretomes (both isolates, Fig 2F and 2G) revealed that the majority of proteins have metabolic functions (152 protein in response to WB and 135 in response to GS) such as protein metabolism (including proteolysis), carbohydrate metabolism (including monosaccharides and polysaccharide metabolism as well as glycolysis), nucleobase-containing compounds metabolic process and lipid metabolism (only WB). The same metabolic groups were also seen in the control Caco-2 cell secretome (S2C Fig). One of the big groups that emerged in our analysis was the cytoskeletal proteins (41 proteins in response to WB isolate and 32 proteins in response to GS). This group included the intermediate filament and the actin family cytoskeletal proteins (including non-motor actin binding protein in the child lineage). Except for the actin family cytoskeletal protein group which was not enriched in control Caco-2 cell secretome, the rest of cytoskeletal protein groups contained more proteins compared to the control secretome. This clearly indicates the effect of *Giardia* colonization on IECs actin cytoskeleton. An interesting finding is that seven microvilli proteins were detected in the secretome of Caco-2 cells incubated with WB trophozoites (GO:0005903, intestinal epithelial brush border) (e.g. villin-1, intelectin-1, myosin heavy chain-9 and -14, beta 1,4- galactosyltransferase polypeptide 1, plectin and LIM domain and actin binding 1). These proteins might have been cleaved off/dissociated from damaged microvilli as a result of trophozoite attachment.

Two of the proteins that we identified in the parasitized Caco-2 cell secretome are associated with immunological functions. The first protein is a cytokine called macrophage migration inhibitory factor (MIF) (Table 3). MIF is a pleiotropic cytokine that regulates the expression of inflammatory genes (e.g. TNF-α, cyclooxygenase 2 and inducible NO synthase (iNOS)) [39], indicating its involvement in innate immune responses. The second protein is complement c3 identified in the secretome of Caco-2 cell incubated with GS only (Table 3), suggesting differences in *Giardia* isolates abilities to activate the complement system.

In conclusion, we have identified the major secreted proteins of host and parasite during interaction. Our analysis showed that both, the host and parasite, release proteins with similar functions (sugar, protein, lipid and nucleic acid metabolism). Both assemblages/isolates (A and B) induced similar responses but there are assemblage-specific differences that might explain observed differences in infectivity and symptoms.

### Transcriptional response of IECs to *Giardia* ESPs

**Fig 3:**
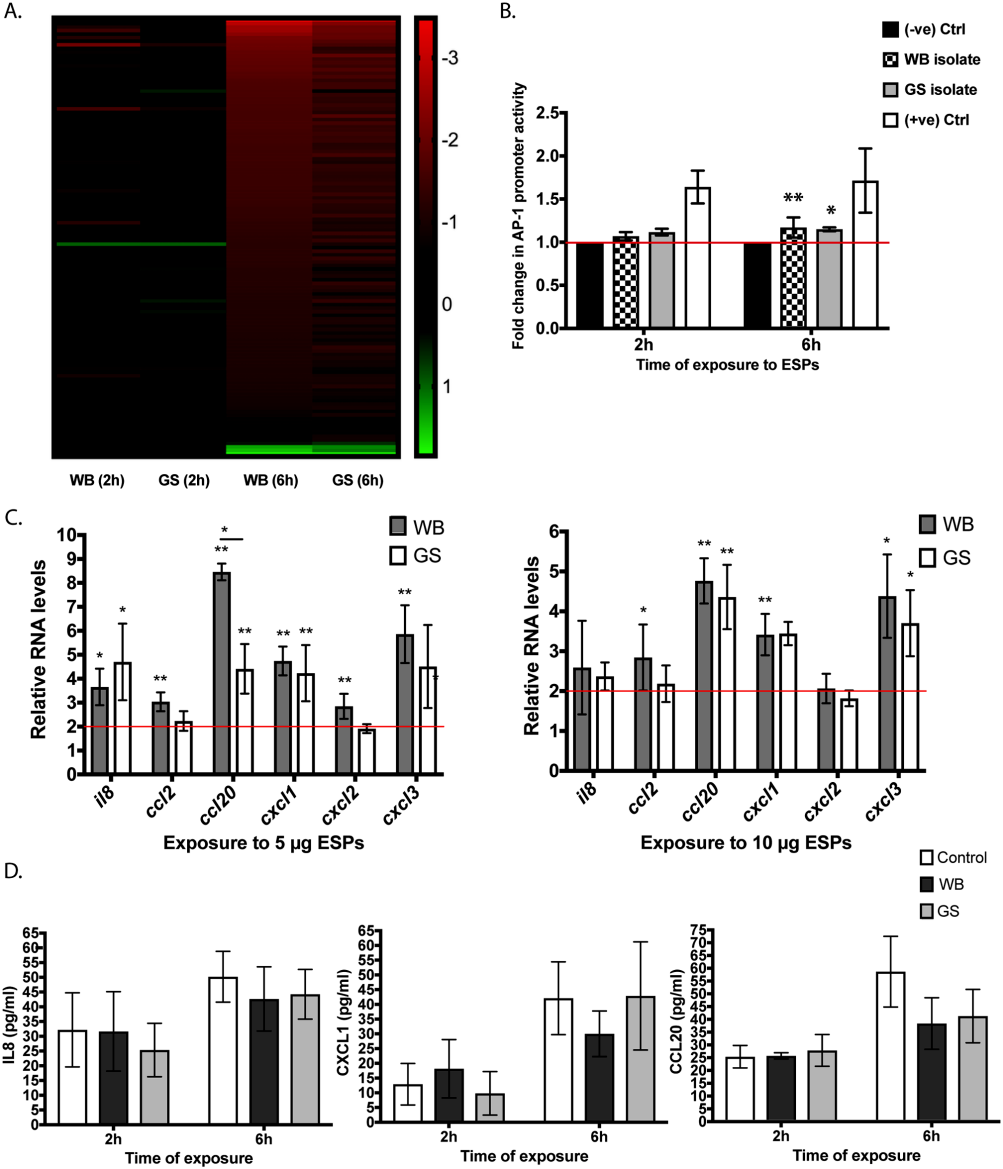
Gene expression changes in IECs after ESP exposure. (A) Heat-map of differentially expressed genes in the differentiated human colon carcinoma cell line Caco-2 exposed to *Giardia intestinalis* excretory secretory products. (B) Induction of AP-1 activity using a luciferase reporter plasmid transfected into Caco-2 cells. The cells were exposed to ESPs of *Giardia intestinalis* isolate WB or GS for 2h or 6h through a Transwell insert placed in the tissue culture plate (Mean ± SD, *n* = 4). (C) Relative RNA levels (Mean ± SD, *n* = 4) of inflammatory genes in differentiated Caco-2 cells incubated with different amounts of ESPs (5μg/ml and 10μg/ml) collected from *Giardia intestinalis* isolate WB or GS. Significant results in the panels are indicated by (*) for *P* < 0.05 or (**) for *P* < 0.01. (D) ELISA measurement (Mean ± SD, *n* = 3) of interleukin-8 (IL-8), chemokine (C-X-C motif) ligand 1 (CXCL1) and chemokine (C-C motif) ligand 20 (CCL20) in the medium of differentiated human colon carcinoma cell line, Caco-2, exposed to ESPs of *G*. *intestinalis* WB or GS isolate through a Transwell insert. No statistical significance was found between controls and treatments (*P* > 0.05).

Interaction between *Giardia* trophozoites and IECs *in vitro* induces the transcription of a large number of genes including the chemokines *ccl2*, *ccl20*, and *cxcl1-3* that are up-regulated up to 100-fold at the RNA level [40]. To test whether *Giardia* ESPs are capable of inducing similar effects, differentiated Caco-2 cells were exposed to *Giardia* ESPs through Transwell inserts (Fig 1B) for 2h and 6h. Differentially expressed genes (DEGs) were identified by RNA sequencing (RNA Seq) (S11 Table). Overall, 120 and 87 genes were differentially expressed (DE) in IECs exposed to ESPs of WB and GS trophozoites, respectively, with a global gene expression profile showing differences between the two time points and between the two isolates (S11 Table, Fig 3A).

At 2h, seven genes were DE in IECs exposed to ESPs of the WB isolate, six of which were up-regulated and one gene (*il8*) was down-regulated (Table 5). The up-regulated genes encode immediate early genes [41], including the protein constituents of AP-1 transcription factor (FBJ murine osteosarcoma viral oncogene homolog, *fos* and jun B proto-oncogene, *junb*). To verify AP-1 activation, Caco-2 cells we transfected with a plasmid expressing a luciferase gene under the control of AP-1 transcription response element and luminescence was measured upon cell exposure to *Giardia* ESPs. At 2h, no significant change was observed in the AP-1 promoter activity with ESPs of either isolates (1.07 for ESPs of WB isolate and 1.12 for ESPs of GS isolate, *P* > 0.05) (Fig 3B). At 6h, on the other hand, despite the slight increase in AP-1 promoter activity (1.17 for ESPs of WB isolate and 1.15 for ESPs of GS isolate), the average fold change was significant (n = 4, *P* < 0.05) (Fig 3B). These findings are in line with the RNA Seq results and indicate that AP-1 is activated in response to *Giardia* ESPs. The gene encoding DUSP1 was the highest up-regulated gene at 2h and the only up-regulated gene in IECs exposed to ESPs of GS trophozoites (Table 5). Its transcriptional level increased at 6h together with dual specificity phosphatases 4 and 5 (*dusp4* and *dusp5*), and tribbles pseudokinase 1 (*trib1*) (Table 5). The products of these genes play important roles in the regulation of MAPKs’ activity [42–44].

**Table 5:** Selected groups of differentially expressed genes in differentiated human colon carcinoma Caco-2 cells exposed to excretory-secretory products of *Giardia intestinalis* isolates WB and GS via Transwell insert. Bold fonts indicate a significantly up-regulated gene whereas italic fonts indicate a significantly down-regulated gene.

Symbol	Gene name	Fold change (2h)		Fold change (6h)	
		WB	GS	WB	GS
*Inhibition of NFκB transcription factor nuclear recruitment*					
**NFKBIA**	nuclear factor of kappa light polypeptide gene enhancer in B-cells inhibitor alpha	1.25	1.05	**3.02**	**2.28**
**BCL3**	B-cell CLL lymphoma 3	1.20	1.19	**2.65**	**2.24**
*Induction of AP-1transcription factor activity*					
**FOS**	FBJ murine osteosarcoma viral oncogene homolog	**2.94**	1.80	**3.04**	**2.45**
**JUNB**	jun B proto-oncogene	**2.28**	1.35	**2.43**	**1.91**
**JUND**	jun D proto-oncogene	1.30	1.07	**3.27**	**2.49**
**FOSB**	FBJ murine osteosarcoma viral oncogene homolog B	1.27	0.97	**3.04**	2.64
*Inhibition of MAPK phosphorylation*					
**DUSP1**	dual specificity phosphatase 1	**4.30**	**2.06**	**4.13**	**3.04**
**DUSP5**	dual specificity phosphatase 5	1.19	0.95	**2.80**	**2.49**
**DUSP4**	dual specificity phosphatase 4	1.06	1.07	2.03	**2.38**
**TRIB1**	tribbles pseudokinase 1	1.37	1.10	**2.79**	**3.02**
*Induction of inflammatory responses*					
**IL8**	interleukin 8	*0*.*52*	*0*.*52*	**2.34**	1.95
**CXCL1**	chemokine C-X-C motif ligand 1 melanoma growth stimulating activity alpha	0.99	0.92	**2.22**	1.23
**CXCL2**	chemokine C-X-C motif ligand 2	1.66	1.41	**3.87**	**2.42**
**CXCL3**	chemokine C-X-C motif ligand 3	1.10	1.20	**4.08**	**2.31**
**CCL2**	chemokine C-C motif ligand 2	0.85	0.79	**2.23**	1.21
**CCL20**	chemokine C-C motif ligand 20	1.14	0.71	**3.13**	0.90
**CLCF1**	cardiotrophin-like cytokine factor 1	1.28	1.05	**2.34**	**2.56**
*Modulation of immune responses via RNA decay*					
**ZFP36**	ZFP36 ring finger protein	**2.27**	1.43	**4.41**	**3.85**
**ZC3H12A**	zinc finger CCCH-type containing 12A*	1.63	1.22	**2.77**	**2.00**
**ZFP36L2**	ZFP36 ring finger protein-like 2	1.38	1.14	**2.02**	**1.94**
*Tight junction proteins*					
**CLDN3**	claudin 3	1.23	1.23	**2.10**	**2.00**
**CLDN4**	claudin 4	1.28	1.09	**2.57**	**2.15**
*Cell cycle regulation*					
**NR4A1**	nuclear receptor subfamily 4 group A member 1*^,^^Ψ^	0.98	0.91	**7.52**	**4.95**
**GADD45A**	growth arrest and DNA-damage-inducible alpha	1.22	1.08	**4.28**	**3.39**
**PLK3**	polo-like kinase 3	1.17	1.02	**2.75**	**2.41**
**DDIT**	DNA-damage-inducible transcript 4*	1.38	1.12	**2.04**	**1.86**
**BTG2**	BTG family member 2	1.97	1.36	**6.03**	**4.01**
*Induction/inhibition of apoptosis*					
**IER3**	immediate early response 3	1.08	0.89	**1.84**	**1.83**
**PIM3**	pim-3 oncogene	1.38	1.14	**2.71**	**2.27**
**PPP1R15A**	protein phosphatase 1 regulatory subunit 15A	1.39	1.06	**2.75**	**2.25**
**BIK**	BCL2-interacting killer apoptosis-inducing	1.05	1.04	**2.22**	**2.49**
*Glucose uptake and response to glucose starvation*					
**INSIG1**	Insulin induced gene 1	0.99	0.83	**11.21**	**8.55**
**NUAK2**	NUAK family SNF1-like kinase 2**	1.23	0.99	**2.14**	**1.64**
**SLC2A1**	solute carrier family 2 facilitated glucose transporter member 1	1.12	1.11	**1.72**	1.60
**SLC2A3**	solute carrier family 2 facilitated glucose transporter member 3	1.07	0.86	**1.50**	**1.61**

*other function; promotes apoptosis,

**other function; anti-apoptotic,

^Ψ^ anti-inflammatory

The majority of DEGs (120 in WB and 87 in GS) were up-regulated at 6h (S11 Table) with a higher fold change for DEGs compared to 2h (Table 5, S11 Table). One exception was that the increase in RNA levels *il8* in of IECs exposed to GS trophozoite (1.95 fold) was not significant. Insulin induced gene 1 (*insig1*) was the highest up-regulated gene in IECs in response to ESPs of both isolates at 6h (Table 5) and this gene encodes a protein that regulates cholesterol metabolism, lipogenesis, and glucose homeostasis [45–47]. More genes associated with glucose uptake and response to glucose starvation (Table 5) were DE at 6h, indicating that *Giardia* releases ESPs that interfere with glucose homeostasis. Nuclear receptor subfamily 4 group A member 1 (*nr4a1*) was the second top up-regulated gene (ESPs of both isolates, Table 5) and this gene has functions associated with cell cycle, inflammation, and induction of apoptosis [48–50]. Another two cell cycle genes associated with response to DNA damage, were amongst the top ten up-regulated genes and these include BTG family member 2 (*btg2*) [51] and growth arrest and DNA damage inducible alpha (*gadd45a*) [52] (ESPs of both isolates, Table 5). This suggests that *Giardia* ESPs induce damaging effects on host DNA and arrests IECs in the cell cycle. The tight junction proteins, claudin 3 and 4 (*cldn 3* and *cldn 4*), were also up-regulated (Table 5) and these proteins play essential roles in the maintenance of the intestinal epithelial barrier (IEB) [53].

To obtain a general overview on the function of DEGs, they were processed through the GO Database for biological and molecular functions. For IECs exposed to ESPs of the WB isolate, cell proliferation (GO:0008283) and MAPK cascade (GO:0000165) constituted the biggest biological groups encompassing 11 genes each. Both chemokines (e.g. CXCL1-3 and IL-8) and growth factors (e.g. amphiregulin, proepiregulin, Proheparin-binding EGF-like growth factor) were contained within the cell proliferation group. All chemokine genes, including *ccl2* and *ccl20*, were listed under chemokine activity (GO:0008009) and cytokine receptor binding (GO:0005126) by molecular function. In addition to these cytokines, the gene *clcf1* encoding cardiotrophin-like_cytokine_factor_1 was DE in IECs exposed to ESPs of WB trophozoites. CLCF1 is an IL-6 family cytokine capable of activating NFκB and exerts stimulatory functions on B cells [54]. For MAPK cascade, it was interesting to see genes encoding RNA binding proteins (*zfp36* and *zfp36l2*) within this group, both of which are associated with anti-inflammatory functions [55]. Another DE gene is Regnase 1 (ZC3H12A), an RNase that controls inflammatory responses by inducing mRNA decay of specific cytokine transcripts [56]. The rest of functional groups in this analysis included response to external stimulus (GO:0009605), locomotion (GO:0040011), localization (GO:0051179), behaviour (GO:0007610) and cell death (GO:0008219). For IECs exposed to ESPs of the GS isolate, the groups cell proliferation, MAPK cascade, cell death and endoderm development (GO:0007492) emerged by biological functions and no specific groups emerged by molecular function analysis. Only *cxcl2*, *cxcl3* and *clcf1* were significantly induced in IECs exposed to ESPs of the GS trophozoites. Overall, based on the transcriptomic profile we could identify a general response in IECs exposed to *Giardia* ESPs, which involves inducing and regulating inflammatory responses, glucose homeostasis, apoptosis, cell cycle, and maintenance of IEB.

Next, we examined which amount of ESPs could induce the transcription of pro-inflammatory genes in IECs. ESPs collected from axenic culture (1, 5 and 10 μg/ml) were incubated with IECs for 2h or 6h (Fig 3C) and RNA levels were assessed with qPCR. At 2h, 5 μg/ml of WB isolate ESPs induced a 2 to 2.9-fold increase in RNA levels of *il8*, *ccl20*, and *cxcl1-3* but not *ccl2*, scoring the highest for *cxcl3* (Fig 3C). The fold change, however, was statistically insignificant for all the genes tested (*P* > 0.05). The fold change in RNA levels remained below 2 for 1 μg/ml (not shown) and 10 μg/ml of ESPs (Fig 3C). At 6h, all genes were significantly up-regulated (2.84–8.46 fold) in IECs incubated with 5 μg/ml WB isolate ESPs, scoring the highest for *ccl20* (*P* < 0.05) and the genes *ccl2*, *ccl20*, *cxcl1* and *cxcl3* remained significantly up-regulated (2.84–4.76 fold) when IECs were incubated with 10 μg/ml of ESPs (Fig 3C). For ESPs of the GS isolate, the fold change at RNA level for all the genes tested at the three concentrations of ESPs was below 2 (*P* > 0.05) (Fig 3D). Nevertheless, at 6h, *il8*, *ccl20*, *cxcl1* and *cxcl3* were significantly up-regulated (4.23–4.71 fold) with *il8* being significantly up-regulated at all ESPs concentrations tested and *ccl20* and *cxcl1* at 10 μg/ml (Fig 3C). The results show that ESPs of both isolates induce variable transcriptional responses of inflammatory genes in IECs and these responses differ with the amounts of ESPs IECs are exposed to. However, direct host-cell interactions have a much stronger effect on the induction of chemokine gene transcription [40].

To assess whether the up-regulated chemokine genes were translated into their protein products, we selected three chemokines (IL-8, CXCL1 and CCL20) and measured their levels in the collected media from the insert experiment (Fig 3D). The amounts of IL8, CXCL1 and CCL20 were very low and their concentration was similar or lower than their controls at 6h (50.2, 42.1 and 57.9 pg/ml, respectively). A slight increase in the amounts of CXCL1 and CCL20 amounts could be seen at 2h upon IECs exposure to ESPs of the WB trophozoites (12.9 pg/ml in control versus 18.2 pg/ml) and GS trophozoites (24.7 pg/ml in control versus 27.1 pg/ml), respectively (*P* > 0.05) (Fig 3D). Overall, the levels of measured chemokines were either close to control or slightly lower and no significant differences could be seen between IECs exposed or unexposed to ESPs (*P* > 0.05) (Fig 3D). The low levels of measured chemokines indicate that their mRNA levels are post-transcriptionally regulated or the chemokines being degraded upon release from IECs.

### The effect of ESPs on cell signaling

**Fig 4:**
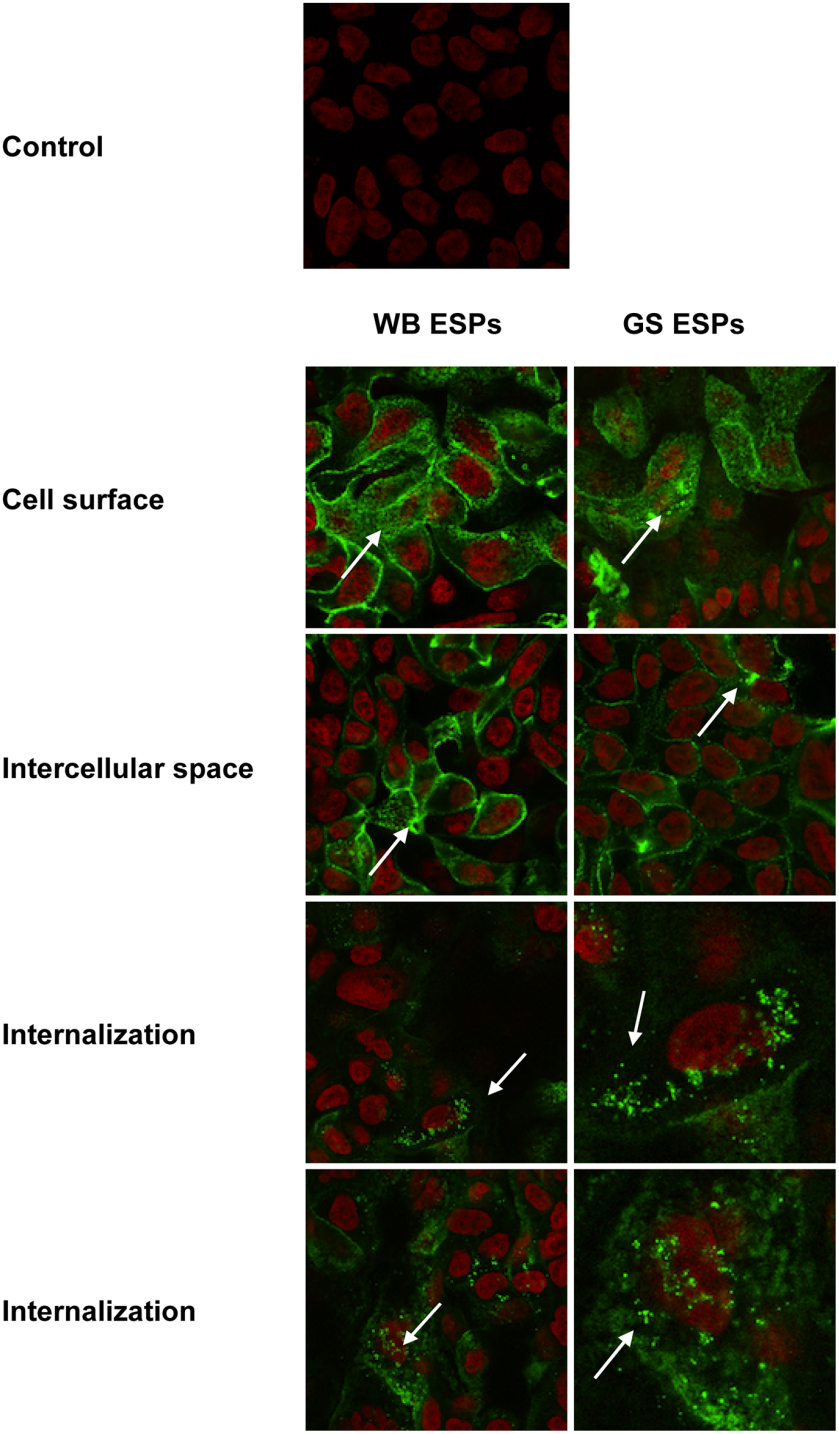
The binding and internalization of *Giardia intestinalis* excreted-secreted products (ESPs) to intestinal epithelial cells (IECs). Confocal microscopy images of differentiated human colon carcinoma cell line, Caco-2, incubated with Alexa Fluor488-labelled ESPs of *G*. *intestinalis* isolate WB or GS. Note that the colour of nucleus, stained with DAPI, was changed to red for better contrast.

*Giardia* ESPs from axenic cultures were labelled with Alexa Fluor488 and added to the medium to visualize their interaction with IECs. Confocal images taken upon 2h and 6h of incubation with IECs showed that the labelled ESPs of WB and GS trophozoites bind to the IECs surface as well as the intercellular space (i.e. intercellular junctions) and some are internalized into differentiated Caco-2 cells (Fig 4). This indicates the presence of ligands or receptors on IECs surface and a potential of the internalized ESPs to modulate cellular processes and signaling.

**Fig 5:**
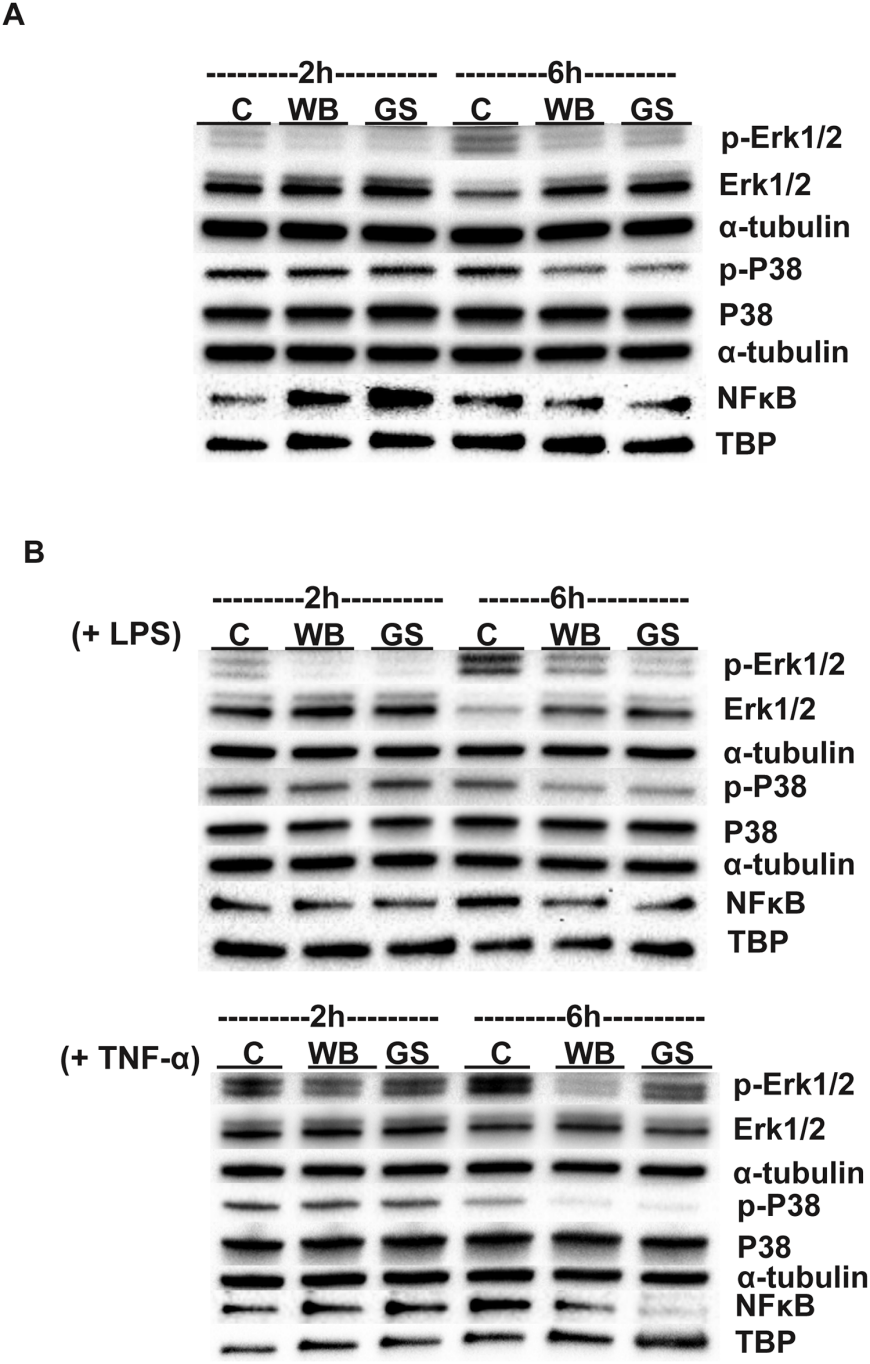
The effects of ESPs on IEC cell signaling. (A) Western blot analyses of differentiated Caco-2 cells exposed to *G*. *intestinalis* ESPs through a Transwell insert for 2h or 6h in the absence of inflammatory stimuli (e.g. lipopolysaccharides or tumour necrosis factor-alpha). Blots were probed for total proteins (phosphorylated/non-phosphorylated forms) of mitogen activated proteins kinases (MAPKs, ERK1/2 and P38) and nuclear factor kappa beta (NFκB). (B) Western blot analyses after the induction of inflammation with LPS and TNF-alpha.

We consequently examined MAPK signaling in IECs exposed to ESPs via inserts because this pathway emerged in the functional analysis of RNA Seq data. Western blot analyses showed a slight decrease in the phosphorylation levels of ERK1/2 and P38 at 2h but an increased level of nuclear translocation of NFκB compared to control (Fig 5A). Despite the slight decrease in ERK1/2 and P38 phosphorylation, their roles in inducing the nuclear recruitment of NFκB cannot be excluded and it indicates a possibility of other factors contributing to NFκB activation (e.g. growth factors, stress, hypoxia or nutrient depletion). At 6h, we could see a marked reduction in the phosphorylation levels of ERK1/2 and P38 compared to control and this coincided with a decrease in the levels of NFκB nuclear translocation (Fig 5A). The nuclear translocation of NFκB, however, was not completely abolished. The phosphorylated form of JNK could not be detected (S3A Fig). This shows that *Giardia* ESPs actively modulate MAPK signaling to avert the induction of strong inflammatory responses and this effect is built up upon prolonged exposure of IECs to ESPs. To test whether *Giardia* ESPs can also inhibit inflammatory signaling, we incubated IECs with LPS or TNF-α for 2h, then added *Giardia* trophozoites into Transwell inserts in the persistence of inflammatory stimuli for 2h or 6h. While the controls exhibited increased phosphorylation of ERK1/2 and P38 as well as nuclear recruitment of NFκB, these levels were reduced in inflamed tissue upon exposure to *Giardia* ESPs at both time points (Fig 5B). These results show that *Giardia* ESPs can attenuate inflammatory signaling mediated by MAPK phosphorylation and NFκB recruitment. It is possible that the parasite senses IECs inflammatory signals and release specific protein factors to subvert such effects.

## Discussion

Several recent studies have shed the light on the secretomes of different protozoan parasites and their role in parasite physiology, adaptation to host environment, pathogenicity and immune modulation [36,37,57–59]. To date, very few *Giardia* ESPs have been identified, and thus we devised experiments to characterise *Giardia* secretome, identify secreted proteins that may be involved in virulence and better understand disease mechanism (Fig 1). We also assessed the effect of *Giardia* colonization on host cells and identified IECs secretory responses.

**Fig 6:**
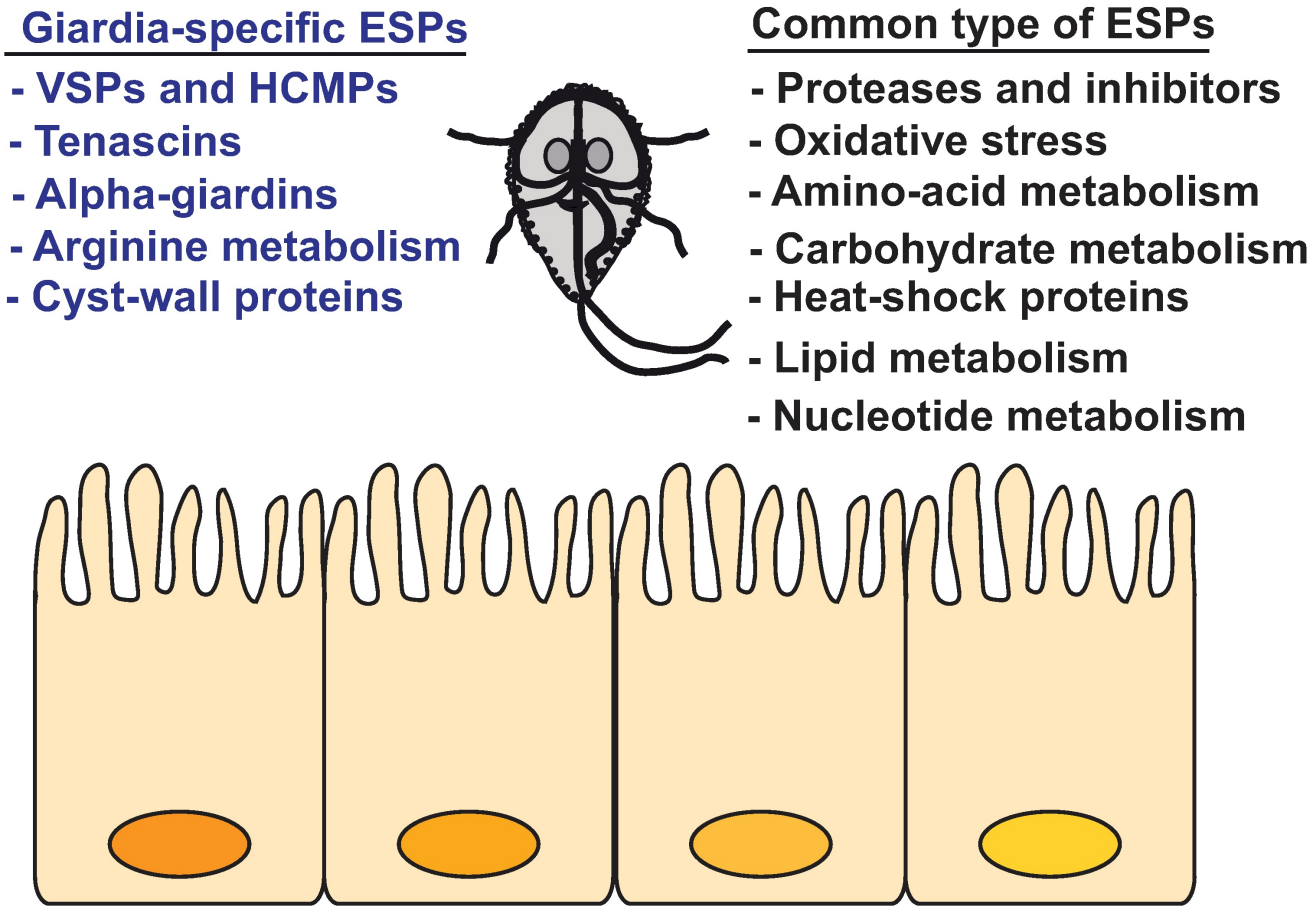
Proteins secreted during *Giardia*-host cell interactions. During *Giardia*-host cell interactions *in vitro* specific proteins are released into the growth medium. The same types of proteins, mainly enzymes, are released from both the host and parasite (black) but there are also parasite-specific proteins being released (blue).

Our analysis highlighted metabolic functions as a dominant secretory response when *Giardia* trophozoites interact with IECs (Fig 6). These functions involve carbohydrate (including glycolysis), protein (including arginine metabolism), lipid (phospholipid remodelling) and nucleic acid (purines and pyrimidines salvage) metabolism. One striking observation is that the same types of metabolic proteins are released by *Giardia* and host cells during interactions, indicating competition for acquiring nutrients (Fig 6).

The release of glycolytic enzymes has been previously reported in *Giardia* [17] and in many parasites [60] including *Trichomonas vaginalis* [61], *T*. *brucei* [36] and *L*. *donovani* [37]. Secreted glycolytic enzymes of parasites have functions associated with virulence, but those of *Giardia* require further investigation.

The protein metabolic group contained proteins similar to those secreted by *L*. *donovani* and *T*. *brucei* [36,37]. In this group, a large number of CPs were identified whose activity is associated with disease induction and immune modulation [12,15,62]. Herein, we report the identity of secreted CPs (Fig 6) as well as other proteases (e.g. serine and metalloproteases). We also showed a variation in protease numbers between the isolates; an aspect underlying virulence [63]. Little is known about the serine and metalloproteases of *Giardia* but studies in *Entamoeba invadens* [64], *Acanthamoeba castellini* [65], *Acanthamoeba lugdunensis* [66] and *T*. *brucei* [67] have identified some roles in differentiation [64,68], pathogenesis (e.g. degradation of ECM and immunoglobulins) [64–67] and invasion [69]. It will be interesting to characterize the function of these secreted proteases during *Giardia* infections.

*Giardia* trophozoites cannot synthesize purines and pyrimidines *de novo* and rely on exogenous sources for their acquisition [70]. UPL-1 is secreted by *Giardia* and its gene is up-regulated during interaction with IECs [33,71]. This enzyme is involved in pyrimidine salvage and it is highly active in the small intestines [72] corroborating the notion of host-parasite competition for nutrients (Fig 6). UPL-1 is also an immunogenic protein recognized by sera from giardiasis patients, indicating its potential as a diagnostic and vaccine target [21]. Interacting *Giardia* and IECs also secrete nucleases but which nucleases’ activity can surpass the other, it is unknown. Nucleases harness nucleic acids for salvaging purines and pyrimidines as reported for *E*. *histolytica* [73], *L*. *donovani* [37] and *T*. *brucie* [36]. Nucleases of *T*. *brucei*, have also been proposed to attenuate nucleic acids-induced inflammatory responses [36].

*Giardia* trophozoites cannot synthesize lipids *de novo* and acquire those from the host [32]. PLB is an enzyme secreted by the parasite and IECs into the interaction medium (Fig 6). PLB is involved in PLs remodeling in *Giardia* and it localizes to brush border membranes of mature enterocytes in humans [74]. In virulent *Candida albicans* strains, PLB is suggested to promote host cell damage and lysis, and the deletion of *plb* attenuated *Candida* virulence in murine models [75]. I3PS is another ESP secreted by *Giardia* and Caco-2 cells. I3PS synthesises I3P, the precursor for all inositol containing compounds including PLs [76]. The deletion of *i3ps* in *Leishmania* renders the parasite avirulent *in vivo* [77] but whether it contributes to *Giardia*’s virulence this requires investigation. Since the above proteins are required for parasite nutrition and thus survival, it will be interesting to test their potential targets for drugs and/or vaccines.

*Giardia* releases proteins with antioxidant functions during growth and interaction with IECs (Fig 6). This highlights the microaerophilic nature of *Giardia* [78] and the mechanism by which the parasite maintains a low redox potential extracellularly leading to enhanced intracellular homeostasis. When readily released by the parasite or in response to interaction with IECs, antioxidant proteins can promptly attenuate host ROS during infection as suggested for *E*. *histolytica* [79–81]. We previously showed that the transcriptional levels of antioxidant genes vary between the WB and GS isolates during oxidative stress [82] and herein we show differences in the array of secreted antioxidant proteins, further pointing out the differences in oxidative stress responses between assemblage A and B *Giardia* parasites.

*Giardia* escapes host and environmental stresses by cysts formation and thus encystation is regarded as a mean of survival for the parasite. In response to IECs, both *Giardia* isolates released proteins involved in cyst wall formation (Fig 6) and these proteins were not detected in axenic culture indicating that host cells provide the signal to initiate encystation. Interestingly, in a recent study that investigated transcriptional changes in bioluminescent WB isolate trophozoites from high density foci in the mice intestines, the authors reported the up-regulation of encystation, oxidative stress and metabolic genes (carbohydrate, protein, lipid and nucleoside base metabolism) [83]. Herein, we show that the protein products for many of up-regulated genes in that study are being released into the interaction medium. Nevertheless, we did not see any cyst formation in our *in vitro* model, indicating that stress alone might not be a sufficient stimuli to complete encystation successfully and possibly requires other factors like bile [84].

*Giardia* VSPs, HCMPs and tenascin were abundant proteins detected in the secretome of both isolates (Fig 6). VSPs are constantly released from trophozoite surface and they are involved in immune evasion during infection [2,20]. HCMPs are similar to VSPs but lack the c-terminal tail. So far, there is no reports on HCMPs function in *Giardia* except for being transcriptionally up-regulated during interaction with IECs [33,40,71]. Tenascins are also up-regulated during interaction with IECs [85]. Despite the lack of true similarity with human tenascins, the presence of an EGF-like domains in these glycoproteins suggests their involvement in protein-protein interaction, cell signaling or adhesion [86,87]. Tenascins are also able to bind lectin [88,89] and thus a role for *Giardia* tenascins in host innate immunity have been suggested previously [85]. The fact that 8 out 10 WB isolate “tenascins” are secreted, glycosylated and up-regulated during interaction with IECs [85] might suggest interesting roles during *Giardia* infection.

Only a small percentage of proteins in the parasite’s secretome were identified with SPP. Recently, several arguments have supported the hypothesis that many parasitic proteins are released in EVs, containing cytosolic and plasma membrane proteins but not proteins from intracellular organelles [60]. In this study, we provided EM evidence on the presence of EVs from *Giardia* trophozoites (S2 Fig). The sorting of EVs cargo, however, is not well understood but the suggestion is that posttranslational modifications (e.g. glycosylation, ubiquitination, phosphorylation or acylation) could target proteins for vesicular secretion [36,60,90]. Whether this is the case in *Giardia*, this requires further investigation.

Functional analysis of parasitized IECs secretome indicated that *Giardia* trophozoites induce cytoskeletal changes in IECs. It is known that *Giardia* adheres to IECs very strongly, leaving marks on cell surface and affecting the organisation of the actin cytoskeleton [91–93]. One interesting finding is that *Giardia* effect on microvilli structure was pronounced by the detection of seven microvilli proteins in the co-culture medium, one of which is villin. Villin plays an essential role in reorganizing microvilli actin filaments as well as actin bundles assembly, capping and severing [94,95]. Villin binds phosphatidylinositol 4,5-bisphosphate (PIP2), enhancing actin bundling and lysophosphatidic acid preventing all actin modifying activities [96,97]. This protein, however, is cleaved during *Giardia* infection, uncoupling its protective role from the actin cytoskeleton [62].

The secretome of parasitized IECs contained host proteins with immunological function. Examples are the MIF and complement factor c3. MIF is a pleiotropic cytokine that induces inflammatory responses mediated by TNF-α, interferon-γ (IFN- γ), IL-1β, IL-12, IL-6 and, IL-8, among others [39]. MIF plays a role in resistance to infection with *Trypanosoma cruzi* [98], *T*. *gondii* [99] *and Leishmania major* [100] but many studies have also reported its contribution in disease pathology [101–103]. It will be interesting to identify its role during *Giardia* infections. The complement factor c3 was specifically released by IECs in response to GS isolate, which was previously shown to activate the complement system [104]. In fact, the complement system contribute to trophozoite killing both *in vitro* and *in vivo* [105,106] and mice with non-functional component system (deficient in mannose-binding lectin 2 or complement factor 3a receptor [107]) exhibit delayed parasite clearance [104]. Therefore, while these results show the induction of innate immune responses by parasitized IECs, it also shows that these responses might vary in infections with different isolates/assemblages.

To our knowledge, this is the first study that has investigated transcriptional changes in IECs exposed to *Giardia* ESPs. Interestingly, transcriptome data brought about and highlighted important pathological processes previously shown to occur during *Giardia* infection. Specifically, the disturbance in glucose homeostasis [108,109] and IEB integrity [91,92], cell cycle arrest [27] and apoptosis [93,110,111]. It could also indicate another pathomechanism that is *Giardia* ESPs might interfere with cholesterol and lipid metabolism based on the differential expression of *insig1* gene. Of note is that transcriptional changes in IECs were not immediate and were only pronounced at 6h, possibly until enough ESPs had accumulated in the media to induce an effect. By comparison, a similar transcriptional profile was seen in parasitized IECs, but transcriptional changes occurred as early as 1.5h upon trophozoite attachment [40]. Therefore, while *Giardia* ESPs could also play a role in disease induction, these effects might be slower and less apparent compared to those induced upon trophozoite attachment to IECs.

*Giardia* ESPs induced the transcription of chemokines genes previously shown to be induced upon trophozoite attachment (e.g. *cxcl1-3*, *ccl2* and *ccl20*) [40]. When produced locally, chemokines attract immune cells (e.g. B cells, T cells, dendritic cells and macrophages) to the infection site [112]. *Giardia* ESPs also induced the transcription of *il8*, an important neutrophils attractant produced during the early phase of infection. NFκB and AP-1 are TFs involved in the induction of inflammatory genes transcription [28,29] and their activity was evident in our results and previously reported by others in response to *Giardia* ESPs [113]. Despite the activation of both TFs, the upstream signaling pathway leading to their activation (i.e. MAPK phosphorylation) appeared to be attenuated but not abolished, even in the presence of inflammatory stimuli. Our transcriptomic data suggests that this attenuation could be mediated by DEGs encoding, *dusp1*, *dusp4*, *dusp5* and *trib1*, all of which are known to regulate/inhibit MAPKs’ activity [42–44,114–116]. Although the results of ERK1/2 and P38 phosphorylation were inconsistent with a previous report [113], these discrepancies might be due to the different experimental setup used to study ESPs effects on MAPK signaling. In our model, IECs were exposed to parasite ESPs released directly into the medium whereas in the other study ESPs were added directly to IECs (HT29). In fact, we got the same result when ESPs collected in the absence of IECs were added directly to Caco-2 cells (S3B Fig). This could suggest that when *Giardia* senses inflammatory signals it actively releases ESPs to attenuate the pathways leading to cytokines production whereas ESPs produced in the absence of IECs exert an opposite effect.

The differential up-regulation of inflammatory cytokines/chemokines genes did not correlate with the amounts of cytokines measured in medium of insert experiment. Indeed, in some instances the levels of IL8, CXCL1 and CCL20 were lower than their controls, indicating degradation of their mRNAs post-transcriptionally or upon synthesis and release into the medium. Tristetraprolin (i.e. *zfp36* or TTP) among DEGs, encodes a protein that binds the AU rich element (ARE) in the 3’UTR of cytokine transcripts inducing their decay and its role in controlling inflammation is well established in the literature [117,118]. Which parasite factors induce TTP transcription are to be identified. Second, in a previous report, *Giardia* ESPs, specifically CPs, were able to degrade IL8 and this has been shown to attenuate neutrophils infiltration into mice intestines even in the presence of inflammatory stimuli [14,15]. Based on our findings it is plausible to propose that ESPs mediate the attenuation of inflammatory responses by promoting the decay of cytokine transcripts and/or cytokines degradation.

To conclude, an interesting picture of host-parasite interactions during giardiasis emerge from this and other studies. Parasites produce ESPs that consist of secreted proteins [11,17,19,85], released surface proteins [20] and EVs [26]. These ESPs affect gene expression, secretion, signaling, metabolism and immune responses in IECs [11,16,40,85,113]. Nevertheless, parasite attachment to IECs induces stronger and more complex responses compared to when IECs are exposed to ESPs only [40]. Concurrently, there is a secretory response by parasitized IECs where similar factors are released affecting parasite attachment, metabolism and gene expression [16,85]. So far, most studies of *Giardia*-host cell interactions have been performed with simplified models using a small selection of human cell lines but it will be important to follow up these results using more complex *in vitro* host systems like enteroids [119]. This can be complemented by *in vivo* experiments of secreted proteins in mice where trophozoites are mixed with early encysting cells [83]. Thus, *Giardia* interaction with IECs is an intricate process and further understanding of this cross-talk could be the key for understanding giardiasis.

## Supporting information

S1 Fig Delineated metabolic pathways associated with the secretome of *Giardia intestinalis* isolates WB and GS identified in axenic culture and the medium of interaction with the intestinal epithelial cells Caco-2 *in vitro*. (TIFF)

S2 Fig (A) GO term analyses of WB and GS trophozoites in axenic culture. (B) Electron micrographs of *Giardia* extracellular vesicles. (C) GO term analyses of differentiated Caco-2 cell secretome in the absence of parasite.
(TIF)

S3 Fig (A) Western blot analyses of differentiated Caco-2 cells exposed to *G*. *intestinalis* ESPs through a Transwell insert for 2h or 6h in the absence of inflammatory stimuli. Blots were probed for the phosphorylated/non-phosphorylated forms of JNK. (B) Western blot analyses of differentiated Caco-2 cells exposed to *G*. *intestinalis* ESPs collected in axenic culture (i.e. in the absence of intestinal epithelial cells). Blots were probed for total proteins (phosphorylated/non-phosphorylated forms) of mitogen activated proteins kinases (MAPKs, ERK1/2 and P38) and nuclear factor kappa beta (NFκB).
(TIF)

S1 Table All proteins identified in the secretome of *Giardia intestinalis* WB isolate in the serum-free medium, RPMI-1640. Proteins are compared between the two time points, 2h and 6h, which are indicated by colours (orange, 2h) and (blue, 6h). Identification criterion for proteins is based on two peptides per protein. Click on (+) sign in each row to see the tryptic peptides identified.
(XLSX)

S2 Table All proteins identified in the secretome of *Giardia intestinalis* GS isolate in the serum-free medium, 1640. Proteins are compared between the two time points, 2h and 6h, which are indicated by colours (orange, 2h) and (blue, 6h). Identification criterion for proteins is based on two matching peptides per protein. Click on (+) sign in each row to see the tryptic peptides identified.
(XLSX)

S3 Table Common and isolate-specific proteins identified in the secretome of *Giardia intestinalis* WB and GS isolates. Open reading frames together with the protein names are presented in columns based on their annotation in the *Giardia* Database.
(XLSX)

S4 Table All secreted proteins identified in in the serum-free medium of the differentiated human colon carcinoma cell line, Caco-2, incubated with *Giardia intestinalis* WB isolate. Proteins are compared between the two time points, 2h and 6h, which are indicated by colours (orange, 2h) and (blue, 6h). Identification criterion is two peptides per protein. Click on the (+) sign in each row to see the tryptic peptides identified.
(XLSX)

S5 Table All secreted proteins identified in in the serum-free medium of the differentiated human colon carcinoma cell line, Caco-2, incubated with *Giardia intestinalis* GS isolate. Proteins are compared between the two time points, 2h and 6h, which are indicated by colours (orange, 2h) and (blue, 6h). Identification criterion is two peptides per protein. Click on the (+) sign in each row to see the tryptic peptides identified.
(XLSX)

S6 Table Immunoreactive proteins of *Giardia* trophozoites during human infection. These proteins are detected as parasite secreted products in the medium of interaction with intestinal epithelial cells *in vitro*.
(XLSX)

S7 Table Proteins identified in *Giardia intestinalis* WB and GS isolates secretome with secretion signal peptide (SSP). (XLSX)

S8 Table All proteins identified in the secretome of differentiated colon carcinoma human cell line (Caco2) in the serum-free medium, DMEM. Proteins are compared between the two time points, 2h and 6h, which are indicated by colours (orange, 2h) and (blue, 6h). Identification criteria for proteins are based on two peptides per protein. Click on the (+) sign in each row to see the tryptic peptides identified.
(XLSX)

S9 Table Secreted proteins of the differentiated human colonic epithelial cell line, Caco2, incubated alone or with *Giardia intestinalis* WB isolate in a serum-free medium. Proteins are listed in columns with headings indicating the time point they were detected.
(XLSX)

S10 Table Secreted proteins of the differentiated human colonic epithelial cell line, Caco2, incubated alone or with *Giardia intestinalis* GS isolate in a serum-free medium. Proteins are listed in columns with headings indicating the time point they were detected.
(XLSX)

S11 Table Transcriptional profile of differentiated human colon carcinoma cell line Caco-2 exposed to excretory-secretory products (ESPs) of *Giardia intestinalis* isolates WB and GS through a Transwell insert placed into tissue culture plates. The table shows transcriptional changes including fold change of genes transcriptions in Caco-2 cells exposed to ESPs of WB or GS isolate for 2h or 6h. The table also contains differentially expressed genes, which are in blue fonts (up-regulated) or red fonts (down-regulated) to indicate their transcriptional activity.
(XLSX)

## References

[1] Einarsson E, Ma’ayeh S, Svärd SG. An up-date on *Giardia* and giardiasis. Curr Opin Microbiol. 2016;34: 47–52. doi: 10.1016/j.mib.2016.07.019 27501461 10.1016/j.mib.2016.07.019

[2] Ankarklev J, Jerlström-Hultqvist J, Ringqvist E, Troell K, Svärd SG. Behind the smile: cell biology and disease mechanisms of *Giardia* species. Nat Rev Microbiol. 2010;8: 413–22. doi: 10.1038/nrmicro2317 20400969 10.1038/nrmicro2317

[3] Cotton JA, Beatty JK, Buret AG. Host parasite interactions and pathophysiology in *Giardia* infections. Int J Parasitol. 2011;41: 925–933. doi: 10.1016/j.ijpara.2011.05.002 21683702 10.1016/j.ijpara.2011.05.002

[4] Rogawski ET, Bartelt LA, Platts-Mills JA, Seidman JC, Samie A, Havt A, et al Determinants and Impact of Giardia Infection in the First 2 Years of Life in the MAL-ED Birth Cohort. J Pediatric Infect Dis Soc. 2017;6: 153–160. doi: 10.1093/jpids/piw082 28204556 10.1093/jpids/piw082 PMC5907871

[5] Halliez MCM, Buret AG. Extra-intestinal and long term consequences of *Giardia duodenalis* infections [Internet]. World Journal of Gastroenterology 2013 pp. 8974–8985. doi: 10.3748/wjg.v19.i47.8974 24379622 10.3748/wjg.v19.i47.8974 PMC3870550

[6] Samra HK, Ganguly NK, Garg UC, Goyal J, Mahajan RC. Effect of excretory-secretory products of *Giardia lamblia* on glucose and phenylalanine transport in the small intestine of Swiss albino mice. Biochem Int. 1988;17: 801–12. Available: http://www.ncbi.nlm.nih.gov/pubmed/3254161 3254161

[7] Jiménez JC, Fontaine J, Grzych J-M, Dei-Cas E, Capron M. Systemic and mucosal responses to oral administration of excretory and secretory antigens from *Giardia intestinalis*. Clin Diagn Lab Immunol. 2004;11: 152–60. Available: http://www.ncbi.nlm.nih.gov/pubmed/14715563 doi: 10.1128/CDLI.11.1.152-160.2004 14715563 10.1128/CDLI.11.1.152-160.2004 PMC321332

[8] Jiménez JC, Morelle W, Michalsky J-C, Dei-Cas E. Excreted/secreted glycoproteins of G. intestinalis play an essential role in the antibody response. Parasitol Res. Springer-Verlag; 2007;100: 715–720. doi: 10.1007/s00436-006-0339-0 17171569 10.1007/s00436-006-0339-0

[9] Shant J, Bhattacharyya S, Ghosh S, Ganguly NK, Majumdar S. A potentially important excretory-secretory product of *Giardia lamblia*. Exp Parasitol. 2002;102: 178–186. doi: 10.1016/S0014-4894(03)00054-7 12856314 10.1016/s0014-4894(03)00054-7

[10] Kaur H, Ghosh S, Samra H, Vinayak VK, Ganguly NK. Identification and characterization of an excretory-secretory product from *Giardia lamblia*. Parasitology. 2001;123: 347–56. doi: 10.1017/S0031182001008629 11676366 10.1017/s0031182001008629

[11] Rodríguez-Fuentes GB, Cedillo-Rivera R, Fonseca-Liñán R, Argüello-García R, Muñoz O, Ortega-Pierres G, et al *Giardia duodenalis*: Analysis of secreted proteases upon trophozoite-epithelial cell interaction in vitro. Mem Inst Oswaldo Cruz. 2006;101: 693–696. doi: 10.1590/S0074-02762006000600020 17072486 10.1590/s0074-02762006000600020

[12] Piña-Vázquez C, Reyes-López M, Ortíz-Estrada G, de la Garza M, Serrano-Luna J. Host-Parasite Interaction: Parasite-Derived and -Induced Proteases That Degrade Human Extracellular Matrix. J Parasitol Res. 2012;2012: 1–24. doi: 10.1155/2012/748206 22792442 10.1155/2012/748206 PMC3390111

[13] Parenti DM. Characterization of a thiol proteinase in *Giardia lamblia*. J Infect Dis. 1989;160: 1076–80. Available: http://www.ncbi.nlm.nih.gov/pubmed/2584755 2584755 10.1093/infdis/160.6.1076

[14] Cotton JA, Motta J-PP, Schenck LP, Hirota SA, Beck PL, Buret AG. *Giardia duodenalis* infection reduces granulocyte infiltration in an in vivo model of bacterial toxin-induced colitis and attenuates inflammation in human intestinal tissue. PLoS One. 2014;9: e109087 doi: 10.1371/journal.pone.0109087 25289678 10.1371/journal.pone.0109087 PMC4188619

[15] Cotton JA, Bhargava A, Ferraz JG, Yates RM, Beck PL, Buret AG. *Giardia duodenalis* cathepsin B proteases degrade intestinal epithelial interleukin-8 and attenuate interleukin-8-induced neutrophil chemotaxis. Infect Immun. 2014;82: 2772–87. doi: 10.1128/IAI.01771-14 24733096 10.1128/IAI.01771-14 PMC4097641

[16] Stadelmann B, Hanevik K, Andersson MK, Bruserud O, Svärd SG. The role of arginine and arginine-metabolizing enzymes during *Giardia*—host cell interactions in vitro. BMC Microbiol. 2013;13: 256 doi: 10.1186/1471-2180-13-256 24228819 10.1186/1471-2180-13-256 PMC4225669

[17] Ringqvist E, Palm JEED, Skarin H, Hehl AB, Weiland M, Davids BJ, et al Release of metabolic enzymes by *Giardia* in response to interaction with intestinal epithelial cells. Mol Biochem Parasitol. 2008;159: 85–91. doi: 10.1016/j.molbiopara.2008.02.005 18359106 10.1016/j.molbiopara.2008.02.005 PMC3658456

[18] Banik S, Viveros PR, Seeber F, Klotz C, Ignatius R, Aebischer T. *Giardia duodenalis* arginine deiminase modulates the phenotype and cytokine secretion of human dendritic cells by depletion of arginine and formation of ammonia. Infect Immun. 2013;81: 2309–2317. doi: 10.1128/IAI.00004-13 23589577 10.1128/IAI.00004-13 PMC3697621

[19] Skarin H, Ringqvist E, Hellman U, Svärd SG. Elongation factor 1-alpha is released into the culture medium during growth of *Giardia intestinalis* trophozoites. Exp Parasitol. 2011;127: 804–810. doi: 10.1016/j.exppara.2011.01.006 21276445 10.1016/j.exppara.2011.01.006

[20] Papanastasiou P, Hiltpold A, Bommeli C, Köhler P. The release of the variant surface protein of *Giardia* to its soluble isoform is mediated by the selective cleavage of the conserved carboxy-terminal domain. Biochemistry. 1996;35: 10143–8. doi: 10.1021/bi960473b 8756478 10.1021/bi960473b

[21] Palm JED, Weiland ME-L, Griffiths WJ, Ljungström I, Svärd SG. Identification of immunoreactive proteins during acute human giardiasis. J Infect Dis. 2003;187: 1849–59. doi: 10.1086/375356 12792861 10.1086/375356

[22] Prucca CG, Slavin I, Quiroga R, Elías E V, Rivero FD, Saura A, et al Antigenic variation in *Giardia lamblia* is regulated by RNA interference. Nature. 2008;456: 750–4. doi: 10.1038/nature07585 19079052 10.1038/nature07585

[23] Faso C, Hehl AB. Membrane trafficking and organelle biogenesis in *Giardia lamblia*: use it or lose it. Int J Parasitol. 2011;41: 471–80. doi: 10.1016/j.ijpara.2010.12.014 21296082 10.1016/j.ijpara.2010.12.014

[24] Evans-Osses I, Reichembach LH, Ramirez MI, Ingrid Evans-Osses1,2 & Luis H. Reichembach2 & Marcel I. Ramirez1 2. Exosomes or microvesicles? Two kinds of extracellular vesicles with different routes to modify protozoan-host cell interaction. Parasitol Res. Springer Berlin Heidelberg; 2015;114: 3567–3575. 10.1007/s00436-015-4659-9 26272631

[25] Schorey JS, Cheng Y, Singh PP, Smith VL. Exosomes and other extracellular vesicles in host-pathogen interactions. EMBO Rep. 2015;16: 24–43. doi: 10.15252/embr.201439363 25488940 10.15252/embr.201439363 PMC4304727

[26] Evans-Osses I, Mojoli A, Monguió-Tortajada M, Marcilla A, Aran V, Amorim M, et al Microvesicles released from *Giardia intestinalis* disturb host-pathogen response in vitro. Eur J Cell Biol. 2017;96: 131–142. doi: 10.1016/j.ejcb.2017.01.005 28236495 10.1016/j.ejcb.2017.01.005

[27] Stadelmann B, Merino MC, Persson L, Svärd SG. Arginine Consumption by the Intestinal Parasite *Giardia intestinalis* Reduces Proliferation of Intestinal Epithelial Cells. PLoS One. 2012;7: e45325 doi: 10.1371/journal.pone.0045325 23028934 10.1371/journal.pone.0045325 PMC3446895

[28] Fujioka S, Niu J, Schmidt C, Sclabas GM, Peng B, Uwagawa T, et al NF-kappaB and AP-1 connection: mechanism of NF-kappaB-dependent regulation of AP-1 activity. Mol Cell Biol. American Society for Microbiology (ASM); 2004;24: 7806–19. doi: 10.1128/MCB.24.17.7806-7819.2004 10.1128/MCB.24.17.7806-7819.2004 PMC507000 15314185

[29] Karin M. The regulation of AP-1 activity by mitogen-activated protein kinases. J Biol Chem. American Society for Biochemistry and Molecular Biology; 1995;270: 16483–6. doi: 10.1074/JBC.270.28.16483 10.1074/jbc.270.28.16483 7622446

[30] Whitmarsh AJ. Regulation of gene transcription by mitogen-activated protein kinase signaling pathways. Biochim Biophys Acta—Mol Cell Res. 2007;1773: 1285–1298. doi: 10.1016/j.bbamcr.2006.11.011 17196680 10.1016/j.bbamcr.2006.11.011

[31] Vallabhapurapu S, Karin M. Regulation and Function of NF-κB Transcription Factors in the Immune System. Annu Rev Immunol. Annual Reviews; 2009;27: 693–733. doi: 10.1146/annurev.immunol.021908.132641 19302050 10.1146/annurev.immunol.021908.132641

[32] Yichoy M, Duarte TT, De Chatterjee A, Mendez TL, Aguilera KY, Roy D, et al Lipid metabolism in *Giardia*: a post-genomic perspective. Parasitology. NIH Public Access; 2011;138: 267–78. doi: 10.1017/S0031182010001277 20880419 10.1017/S0031182010001277 PMC3132189

[33] Ma’ayeh SY, Brook-Carter PT. Representational difference analysis identifies specific genes in the interaction of *Giardia duodenalis* with the murine intestinal epithelial cell line, IEC-6. Int J Parasitol. 2012;42: 501–509. doi: 10.1016/j.ijpara.2012.04.004 22561399 10.1016/j.ijpara.2012.04.004

[34] Davis CG. The many faces of epidermal growth factor repeats. New Biol. 1990;2: 410–9. Available: http://europepmc.org/abstract/MED/2288911 2288911

[35] Ratner DM, Cui J, Steffen M, Moore LL, Robbins PW, Samuelson J. Changes in the N-glycome, glycoproteins with Asn-linked glycans, of *Giardia lamblia* with differentiation from trophozoites to cysts. Eukaryot Cell. American Society for Microbiology; 2008;7: 1930–40. doi: 10.1128/EC.00268-08 18820077 10.1128/EC.00268-08 PMC2583543

[36] Geiger A, Hirtz C, Bécue T, Bellard E, Centeno D, Gargani D, et al Exocytosis and protein secretion in *Trypanosoma*. 10.1186/1471-2180-10-20 PMC3224696 20102621

[37] Silverman JM, Chan SK, Robinson DP, Dwyer DM, Nandan D, Foster LJ, et al Proteomic analysis of the secretome of *Leishmania donovani*. Genome Biol. 2008;9: R35 doi: 10.1186/gb-2008-9-2-r35 18282296 10.1186/gb-2008-9-2-r35 PMC2374696

[38] Marcilla A, Martin-Jaular L, Trelis M, De Menezes-Neto A, Osuna A, Bernal D, et al Extracellular vesicles in parasitic diseases. Int Heal Res. 2014; doi: 10.3402/jev.v3.25040 25536932 10.3402/jev.v3.25040 PMC4275648

[39] Rosado J de D, Rodriguez-Sosa M. Macrophage migration inhibitory factor (MIF): a key player in protozoan infections. Int J Biol Sci. 2011;7: 1239–56. Available: http://www.ncbi.nlm.nih.gov/pubmed/22110378 22110378 10.7150/ijbs.7.1239 PMC3221362

[40] Roxström-Lindquist K, Ringqvist E, Palm D, Svärd S. *Giardia lamblia*-induced changes in gene expression in differentiated caco-2 human intestinal epithelial cells. Infect Immun. 2005;73: 8204–8208. doi: 10.1128/IAI.73.12.8204-8208.2005 16299316 10.1128/IAI.73.12.8204-8208.2005 PMC1307045

[41] Bahrami S, Drabløs F. Gene regulation in the immediate-early response process. Adv Biol Regul. 2016;62: 37–49. doi: 10.1016/j.jbior.2016.05.001 27220739 10.1016/j.jbior.2016.05.001

[42] Wancket LM, Frazier WJ, Liu Y. Mitogen-activated protein kinase phosphatase (MKP)-1 in immunology, physiology, and disease. Life Sci. 2012;90: 237–48. doi: 10.1016/j.lfs.2011.11.017 22197448 10.1016/j.lfs.2011.11.017 PMC3465723

[43] Korhonen R, Turpeinen T, Taimi V, Nieminen R, Goulas A, Moilanen E. Attenuation of the acute inflammatory response by dual specificity phosphatase 1 by inhibition of p38 MAP kinase. Mol Immunol. 2011;48: 2059–68. doi: 10.1016/j.molimm.2011.06.439 21764456 10.1016/j.molimm.2011.06.439

[44] Kiss-Toth E, Bagstaff SM, Sung HY, Jozsa V, Dempsey C, Caunt JC, et al Human tribbles, a protein family controlling mitogen-activated protein kinase cascades. J Biol Chem. American Society for Biochemistry and Molecular Biology; 2004;279: 42703–8. doi: 10.1074/jbc.M407732200 15299019 10.1074/jbc.M407732200

[45] Dong X-Y, Tang S-Q. Insulin-induced gene: A new regulator in lipid metabolism. Peptides. 2010;31: 2145–2150. doi: 10.1016/j.peptides.2010.07.020 20817058 10.1016/j.peptides.2010.07.020

[46] Krapivner S, Chernogubova E, Ericsson M, Ahlbeck-Glader C, Hamsten A, van ‘t Hooft FM. Human evidence for the involvement of insulin-induced gene 1 in the regulation of plasma glucose concentration. Diabetologia. 2007;50: 94–102. doi: 10.1007/s00125-006-0479-x 17106696 10.1007/s00125-006-0479-x

[47] Yang T, Espenshade PJ, Wright ME, Yabe D, Gong Y, Aebersold R, et al Crucial step in cholesterol homeostasis: sterols promote binding of SCAP to INSIG-1, a membrane protein that facilitates retention of SREBPs in ER. Cell. Elsevier; 2002;110: 489–500. doi: 10.1016/S0092-8674(02)00872-3 10.1016/s0092-8674(02)00872-3 12202038

[48] Hamers AAJ, van Dam L, Teixeira Duarte JM, Vos M, Marinković G, van Tiel CM, et al Deficiency of Nuclear Receptor Nur77 Aggravates Mouse Experimental Colitis by Increased NFκB Activity in Macrophages. Moschetta A, editor. PLoS One. Public Library of Science; 2015;10: e0133598 doi: 10.1371/journal.pone.0133598 26241646 10.1371/journal.pone.0133598 PMC4524678

[49] Pei L, Castrillo A, Tontonoz P. Regulation of Macrophage Inflammatory Gene Expression by the Orphan Nuclear Receptor Nur77. Mol Endocrinol. 2006;20: 786–794. doi: 10.1210/me.2005-0331 16339277 10.1210/me.2005-0331

[50] Li H, Kolluri SK, Gu J, Dawson MI, Cao X, Hobbs PD, et al Cytochrome c Release and Apoptosis Induced by Mitochondrial Targeting of Nuclear Orphan Receptor TR3. Science (80-). 2000;289 Available: http://science.sciencemag.org/content/289/5482/1159.long 10.1126/science.289.5482.1159 10947977

[51] Rouault J-P, Falette N, Guéhenneux F, Guillot C, Rimokh R, Wang Q, et al Identification of BTG2, an antiproliferative p53–dependent component of the DNA damage cellular response pathway. Nat Genet. Nature Publishing Group; 1996;14: 482–486. doi: 10.1038/ng1296-482 8944033 10.1038/ng1296-482

[52] Smith M, Chen I, Zhan Q, Bae I, Chen C, Gilmer T, et al Interaction of the p53-regulated protein Gadd45 with proliferating cell nuclear antigen. Science (80-). 1994;266 Available: http://science.sciencemag.org/content/266/5189/1376.long 10.1126/science.7973727 7973727

[53] Lu Z, Ding L, Lu Q, Chen Y-H. Claudins in intestines: Distribution and functional significance in health and diseases. Tissue barriers. Taylor & Francis; 2013;1: e24978 doi: 10.4161/tisb.24978 24478939 10.4161/tisb.24978 PMC3879173

[54] Senaldi G, Varnum BC, Sarmiento U, Starnes C, Lile J, Scully S, et al Novel neurotrophin-1/B cell-stimulating factor-3: a cytokine of the IL-6 family. Proc Natl Acad Sci U S A. National Academy of Sciences; 1999;96: 11458–63. doi: 10.1073/PNAS.96.20.11458 10.1073/pnas.96.20.11458 PMC18055 10500198

[55] Brooks SA, Blackshear PJ. Tristetraprolin (TTP): Interactions with mRNA and proteins, and current thoughts on mechanisms of action. Biochim Biophys Acta—Gene Regul Mech. 2013;1829: 666–679. doi: 10.1016/j.bbagrm.2013.02.003 23428348 10.1016/j.bbagrm.2013.02.003 PMC3752887

[56] Matsushita K, Takeuchi O, Standley DM, Kumagai Y, Kawagoe T, Miyake T, et al Zc3h12a is an RNase essential for controlling immune responses by regulating mRNA decay. Nature. 2009;458: 1185–1190. doi: 10.1038/nature07924 19322177 10.1038/nature07924

[57] Wong WK, Tan ZN, Othman N, Lim BH, Mohamed Z, Olivos Garcia A, et al Analysis of *Entamoeba histolytica* excretory-secretory antigen and identification of a new potential diagnostic marker. Clin Vaccine Immunol. 2011;18: 1913–7. doi: 10.1128/CVI.05356-11 21918120 10.1128/CVI.05356-11 PMC3209014

[58] Song M-J, Lee J-J, Nam YH, Kim T-G, Chung YW, Kim M, et al Modulation of dendritic cell function by *Trichomonas vaginalis*-derived secretory products. BMB Rep. 2015;48: 103–8. Available: http://www.ncbi.nlm.nih.gov/pubmed/24965578 doi: 10.5483/BMBRep.2015.48.2.116 24965578 10.5483/BMBRep.2015.48.2.116 PMC4352611

[59] Grébaut P, Chuchana P, Brizard J-P, Demettre E, Seveno M, Bossard G, et al Identification of total and differentially expressed excreted-secreted proteins from *Trypanosoma congolense* strains exhibiting different virulence and pathogenicity. Int J Parasitol. 2009;39: 1137–50. doi: 10.1016/j.ijpara.2009.02.018 19285981 10.1016/j.ijpara.2009.02.018

[60] Gómez-Arreaza A, Acosta H, Quiñones W, Concepción JL, Michels PAM, Avilán L. Extracellular functions of glycolytic enzymes of parasites: unpredicted use of ancient proteins. Mol Biochem Parasitol. 2014;193: 75–81. doi: 10.1016/j.molbiopara.2014.02.005 24602601 10.1016/j.molbiopara.2014.02.005

[61] Kucknoor AS, Mundodi V, Alderete JF. The proteins secreted by *Trichomonas vaginalis* and vaginal epithelial cell response to secreted and episomally expressed AP65. Cell Microbiol. 2007;9: 2586–97. doi: 10.1111/j.1462-5822.2007.00979.x 17590165 10.1111/j.1462-5822.2007.00979.x PMC2574865

[62] Bhargava A, Cotton JA, Dixon BR, Gedamu L, Yates RM, Buret AG. *Giardia duodenalis* Surface Cysteine Proteases Induce Cleavage of the Intestinal Epithelial Cytoskeletal Protein Villin via Myosin Light Chain Kinase. PLoS One. Public Library of Science; 2015;10: e0136102 doi: 10.1371/journal.pone.0136102 26334299 10.1371/journal.pone.0136102 PMC4559405

[63] de Carvalho TB, David EB, Coradi ST, Guimarães S. Protease activity in extracellular products secreted in vitro by trophozoites of *Giardia duodenalis*. Parasitol Res. 2008;104: 185–90. doi: 10.1007/s00436-008-1185-z 18797927 10.1007/s00436-008-1185-z

[64] Makioka A, Kumagai M, Kobayashi S, Takeuchi T. Involvement of serine proteases in the excystation and metacystic development of *Entamoeba invadens*. Parasitol Res. 2009;105: 977–987. doi: 10.1007/s00436-009-1478-x 19479279 10.1007/s00436-009-1478-x

[65] Na B-K, Kim J-C, Song C-Y. Characterization and pathogenetic role of proteinase from *Acanthamoeba castellanii*. Microb Pathog. 2001;30: 39–48. doi: 10.1006/mpat.2000.0403 11162184 10.1006/mpat.2000.0403

[66] Kim H-K, Ha Y-R, Yu H-S, Kong H-H, Chung D-I. Purification and characterization of a 33 kDa serine protease from *Acanthamoeba lugdunensis* KA/E2 isolated from a Korean keratitis patient. Korean J Parasitol. 2003;41: 189 doi: 10.3347/kjp.2003.41.4.189 14699259 10.3347/kjp.2003.41.4.189 PMC2717510

[67] Huet G, Richet C, Demeyer D, Bisiau H, Soudan B, Tetaert D, et al Characterization of different proteolytic activities in *Trypanosoma brucei* brucei. Biochim Biophys Acta. 1992;1138: 213–21. Available: http://www.ncbi.nlm.nih.gov/pubmed/1547283 1547283 10.1016/0925-4439(92)90040-t

[68] Bangs JD, Ransom DA, Nimick M, Christie G, Hooper NM. In vitro cytocidal effects on *Trypanosoma brucei* and inhibition of Leishmania major GP63 by peptidomimetic metalloprotease inhibitors. Mol Biochem Parasitol. 2001;114: 111–117. doi: 10.1016/S0166-6851(01)00244-4 11356520 10.1016/s0166-6851(01)00244-4

[69] Hainard A, Tiberti N, Robin X, Ngoyi DM, Matovu E, Enyaru JCK, et al Matrix metalloproteinase-9 and intercellular adhesion molecule 1 are powerful staging markers for human African trypanosomiasis. Trop Med Int Health. 2011;16: 119–26. doi: 10.1111/j.1365-3156.2010.02642.x 20958893 10.1111/j.1365-3156.2010.02642.x

[70] Adam RD. Biology of Giardia lamblia. Clin Microbiol Rev. 2001;14: 447–75. doi: 10.1128/CMR.14.3.447-475.2001 11432808 10.1128/CMR.14.3.447-475.2001 PMC88984

[71] Ferella M, Davids BJ, Cipriano MJ, Birkeland SR, Palm D, Gillin FD, et al Gene expression changes during *Giardia*-host cell interactions in serum-freeh medium. Mol Biochem Parasitol. 2014;197: 21–3. doi: 10.1016/j.molbiopara.2014.09.007 25286381 10.1016/j.molbiopara.2014.09.007

[72] el Kouni MH, el Kouni MM, Naguib FN. Differences in activities and substrate specificity of human and murine pyrimidine nucleoside phosphorylases: implications for chemotherapy with 5-fluoropyrimidines. Cancer Res. 1993;53: 3687–93. Available: http://www.ncbi.nlm.nih.gov/pubmed/8339277 8339277

[73] McGugan GC, Joshi MB, Dwyer DM. Identification and biochemical characterization of unique secretory nucleases of the human enteric pathogen, *Entamoeba histolytica*. J Biol Chem. American Society for Biochemistry and Molecular Biology; 2007;282: 31789–802. doi: 10.1074/jbc.M705975200 17766245 10.1074/jbc.M705975200

[74] Tojo H, Ichida T, Okamoto M. Purification and characterization of a catalytic domain of rat intestinal phospholipase B/lipase associated with brush border membranes. J Biol Chem. 1998;273: 2214–21. Available: http://www.ncbi.nlm.nih.gov/pubmed/9442064 9442064 10.1074/jbc.273.4.2214

[75] Ghannoum MA. Potential role of phospholipases in virulence and fungal pathogenesis. Clin Microbiol Rev. 2000;13: 122–43, table of contents. Available: http://www.ncbi.nlm.nih.gov/pubmed/10627494 10627494 10.1128/cmr.13.1.122-143.2000 PMC88936

[76] Majumder AL, Johnson MD, Henry SA. 1l-myo-Inositol-1-phosphate synthase. Biochim Biophys Acta—Lipids Lipid Metab. 1997;1348: 245–256. doi: 10.1016/S0005-2760(97)00122-7 10.1016/s0005-2760(97)00122-7 9370339

[77] Ilg T. Generation of myo-inositol-auxotrophic *Leishmania mexicana* mutants by targeted replacement of the myo-inositol-1-phosphate synthase gene. Mol Biochem Parasitol. 2002;120: 151–6. Available: http://www.ncbi.nlm.nih.gov/pubmed/11849714 11849714 10.1016/s0166-6851(01)00435-2

[78] Brown DM, Upcroft JA, Edwards MR, Upcroft P. Anaerobic bacterial metabolism in the ancient eukaryote *Giardia duodenalis*. Int J Parasitol. 1998;28: 149–64. Available: http://www.ncbi.nlm.nih.gov/pubmed/9504342 9504342 10.1016/s0020-7519(97)00172-0

[79] Sen A, Chatterjee NS, Akbar MA, Nandi N, Das P. The 29-kilodalton thiol-dependent peroxidase of *Entamoeba histolytica* is a factor involved in pathogenesis and survival of the parasite during oxidative stress. Eukaryot Cell. American Society for Microbiology (ASM); 2007;6: 664–73. doi: 10.1128/EC.00308-06 17307964 10.1128/EC.00308-06 PMC1865653

[80] Cheng X-J, Yoshihara E, Takeuchi T, Tachibana H. Molecular characterization of peroxiredoxin from *Entamoeba moshkovskii* and a comparison with *Entamoeba histolytica*. Mol Biochem Parasitol. 2004;138: 195–203. doi: 10.1016/j.molbiopara.2004.08.009 15555731 10.1016/j.molbiopara.2004.08.009

[81] Choi M-H, Sajed D, Poole L, Hirata K, Herdman S, Torian BE, et al An unusual surface peroxiredoxin protects invasive *Entamoeba histolytica* from oxidant attack. Mol Biochem Parasitol. 2005;143: 80–9. doi: 10.1016/j.molbiopara.2005.04.014 15996766 10.1016/j.molbiopara.2005.04.014

[82] Ma’ayeh SY, Knörr L, Svärd SG. Transcriptional profiling of *Giardia intestinalis* in response to oxidative stress. Int J Parasitol. 2015; doi: 10.1016/j.ijpara.2015.07.005 26341007 10.1016/j.ijpara.2015.07.005

[83] Pham JK, Nosala C, Scott EY, Nguyen KF, Hagen KD, Starcevich HN, et al Transcriptomic Profiling of High-Density Giardia Foci Encysting in the Murine Proximal Intestine. Front Cell Infect Microbiol. Frontiers Media SA; 2017;7: 227 doi: 10.3389/fcimb.2017.00227 28620589 10.3389/fcimb.2017.00227 PMC5450421

[84] Gillin FD, Reiner DS, Boucher SE. Small-intestinal factors promote encystation of *Giardia lamblia in vitro*. Infect Immun. 1988;56: 705–707. 3343054 10.1128/iai.56.3.705-707.1988 PMC259350

[85] Emery SJ, Mirzaei M, Vuong D, Pascovici D, Chick JM, Lacey E, et al Induction of virulence factors in *Giardia duodenalis* independent of host attachment. Sci Rep. Nature Publishing Group; 2016;6: 20765 doi: 10.1038/srep20765 26867958 10.1038/srep20765 PMC4751611

[86] Balzar M, Briaire-de Bruijn IH, Rees-Bakker HA, Prins FA, Helfrich W, de Leij L, et al Epidermal growth factor-like repeats mediate lateral and reciprocal interactions of Ep-CAM molecules in homophilic adhesions. Mol Cell Biol. 2001;21: 2570–80. doi: 10.1128/MCB.21.7.2570-2580.2001 11259604 10.1128/MCB.21.7.2570-2580.2001 PMC86888

[87] Baron M. An overview of the Notch signalling pathway. Semin Cell Dev Biol. 2003;14: 113–9. Available: http://www.ncbi.nlm.nih.gov/pubmed/12651094 12651094 10.1016/s1084-9521(02)00179-9

[88] Aspberg A, Miura R, Bourdoulous S, Shimonaka M, Heinegârd D, Schachner M, et al The C-type lectin domains of lecticans, a family of aggregating chondroitin sulfate proteoglycans, bind tenascin-R by protein-protein interactions independent of carbohydrate moiety. Proc Natl Acad Sci U S A. 1997;94: 10116–21. doi: 10.1073/pnas.94.19.10116 9294172 10.1073/pnas.94.19.10116 PMC23322

[89] Aspberg A. The versican C-type lectin domain recognizes the adhesion protein tenascin-R. Proc Natl Acad Sci U S A. 1995;92: 10590–10594. doi: 10.1073/pnas.92.23.10590 7479846 10.1073/pnas.92.23.10590 PMC40657

[90] Moreno-Gonzalo O, Villarroya-Beltri C, Sánchez-Madrid F. Post-translational modifications of exosomal proteins. Front Immunol. Frontiers Media SA; 2014;5: 383 doi: 10.3389/fimmu.2014.00383 25157254 10.3389/fimmu.2014.00383 PMC4128227

[91] Humen MA, Pérez PF, Liévin-Le Moal V. Lipid raft-dependent adhesion of Giardia intestinalis trophozoites to a cultured human enterocyte-like Caco-2/TC7 cell monolayer leads to cytoskeleton-dependent functional injuries. Cell Microbiol. 2011;13: 1683–1702. doi: 10.1111/j.1462-5822.2011.01647.x 21790940 10.1111/j.1462-5822.2011.01647.x

[92] Maia-Brigagão C, Morgado-Díaz JA, De Souza W. *Giardia* disrupts the arrangement of tight, adherens and desmosomal junction proteins of intestinal cells. Parasitol Int. 2012;61: 280–287. doi: 10.1016/j.parint.2011.11.002 22146155 10.1016/j.parint.2011.11.002

[93] Chin AC, Teoh DA, Scott KG-E, Meddings JB, Macnaughton WK, Buret AG. Strain-dependent induction of enterocyte apoptosis by *Giardia lamblia* disrupts epithelial barrier function in a caspase-3-dependent manner. Infect Immun. 2002;70: 3673–80. Available: http://www.pubmedcentral.nih.gov/articlerender.fcgi?artid=128105&tool=pmcentrez&rendertype=abstract doi: 10.1128/IAI.70.7.3673-3680.2002 12065509 10.1128/IAI.70.7.3673-3680.2002 PMC128105

[94] Khurana S, George SP. Regulation of cell structure and function by actin-binding proteins: Villin’s perspective. FEBS Letters. 2008 pp. 2128–2139. doi: 10.1016/j.febslet.2008.02.040 18307996 10.1016/j.febslet.2008.02.040 PMC2680319

[95] Costa de Beauregard M a, Pringault E, Robine S, Louvard D. Suppression of villin expression by antisense RNA impairs brush border assembly in polarized epithelial intestinal cells. EMBO J. 1995;14: 409–21. Available: http://www.pubmedcentral.nih.gov/articlerender.fcgi?artid=398099&tool=pmcentrez&rendertype=abstract 7859732 10.1002/j.1460-2075.1995.tb07017.x PMC398099

[96] Kumar N, Zhao P, Tomar A, Galea CA, Khurana S. Association of Villin with Phosphatidylinositol 4,5-Bisphosphate Regulates the Actin Cytoskeleton. J Biol Chem. 2004;279: 3096–3110. doi: 10.1074/jbc.M308878200 14594952 10.1074/jbc.M308878200

[97] Tomar A, George SP, Mathew S, Khurana S. Differential effects of lysophosphatidic acid and phosphatidylinositol 4,5-bisphosphate on actin dynamics by direct association with the actin-binding protein villin. J Biol Chem. 2009;284: 35278–35282. doi: 10.1074/jbc.C109.060830 19808673 10.1074/jbc.C109.060830 PMC2790956

[98] Reyes JL, Terrazas LI, Espinoza B, Cruz-Robles D, Soto V, Rivera-Montoya I, et al Macrophage migration inhibitory factor contributes to host defense against acute *Trypanosoma cruzi* infection. Infect Immun. American Society for Microbiology (ASM); 2006;74: 3170–9. doi: 10.1128/IAI.01648-05 16714544 10.1128/IAI.01648-05 PMC1479264

[99] Flores M, Saavedra R, Bautista R, Viedma R, Tenorio EP, Leng L, et al Macrophage migration inhibitory factor (MIF) is critical for the host resistance against *Toxoplasma gondii*. FASEB J. The Federation of American Societies for Experimental Biology; 2008;22: 3661–71. doi: 10.1096/fj.08-111666 18606868 10.1096/fj.08-111666 PMC2537436

[100] Satoskar AR, Bozza M, Rodriguez Sosa M, Lin G, David JR. Migration-Inhibitory Factor Gene-Deficient Mice Are Susceptible to Cutaneous *Leishmania major* Infection. Infect Immun. 2001;69: 906–911. doi: 10.1128/IAI.69.2.906-911.2001 11159984 10.1128/IAI.69.2.906-911.2001 PMC97968

[101] Stijlemans B, Leng L, Brys L, Sparkes A, Vansintjan L, Caljon G, et al MIF contributes to *Trypanosoma brucei* associated immunopathogenicity development. Mitre E, editor. PLoS Pathog. 2014;10: e1004414 doi: 10.1371/journal.ppat.1004414 25255103 10.1371/journal.ppat.1004414 PMC4177988

[102] Cavalcanti MG, Mesquita JS, Madi K, Feijó DF, Assunção-Miranda I, Souza HSP, et al MIF participates in *Toxoplasma gondii*-induced pathology following oral infection. Fritz JH, editor. PLoS One. 2011;6: e25259 doi: 10.1371/journal.pone.0025259 21977228 10.1371/journal.pone.0025259 PMC3178626

[103] Santos-Oliveira JR, Regis EG, Leal CRB, Cunha R V., Bozza PT, Da-Cruz AM. Evidence That Lipopolisaccharide May Contribute to the Cytokine Storm and Cellular Activation in Patients with Visceral Leishmaniasis. Carvalho EM, editor. PLoS Negl Trop Dis. 2011;5: e1198 doi: 10.1371/journal.pntd.0001198 21765960 10.1371/journal.pntd.0001198 PMC3134430

[104] Li E, Tako EA, Singer SM. Complement Activation by *Giardia duodenalis* Parasites through the Lectin Pathway Contributes to Mast Cell Responses and Parasite Control. Appleton JA, editor. Infect Immun. American Society for Microbiology (ASM); 2016;84: 1092–9. doi: 10.1128/IAI.00074-16 26831470 10.1128/IAI.00074-16 PMC4807472

[105] Deguchi M, Gillin FD, Gigli I. Mechanism of killing of *Giardia lamblia* trophozoites by complement. J Clin Invest. 1987;79: 1296–302. doi: 10.1172/JCI112952 3646264 10.1172/JCI112952 PMC424366

[106] Hill DR, Burge JJ, Pearson RD. Susceptibility of *Giardia lamblia* trophozoites to the lethal effect of human serum. J Immunol. 1984;132: 2046–52. Available: http://www.ncbi.nlm.nih.gov/pubmed/6699407 6699407

[107] Dunkelberger JR, Song W-C. Complement and its role in innate and adaptive immune responses. Cell Res. Nature Publishing Group; 2010;20: 34–50. doi: 10.1038/cr.2009.139 20010915 10.1038/cr.2009.139

[108] Yu LCH, Huang C, Kuo W, Sayer H, Turner JR, Buret AG. SGLT-1-mediated glucose uptake protects human intestinal epithelial cells against Giardia duodenalis-induced apoptosis. Int J Parasitol. 2008;38: 923–934. doi: 10.1016/j.ijpara.2007.12.004 18281046 10.1016/j.ijpara.2007.12.004 PMC2693066

[109] Mahmood S, Sodhi CP, Ganguly NK. Expression of sodium-glucose co-transporter and brush border disaccharidases in *Giardia lamblia* infected rat intestine. Indian J Biochem Biophys. 2002;39: 185–90. Available: http://www.ncbi.nlm.nih.gov/pubmed/22905389 22905389

[110] Koh WH, Geurden T, Paget T, O’Handley R, Steuart RF, Thompson RCA, et al *Giardia duodenalis* assemblage-specific induction of apoptosis and tight junction disruption in human intestinal epithelial cells: effects of mixed infections. J Parasitol. 2013;99: 353–8. doi: 10.1645/GE-3021.1 22924932 10.1645/GE-3021.1

[111] Panaro MA, Cianciulli A, Mitolo V, Mitolo CI, Acquafredda A, Brandonisio O, et al Caspase-dependent apoptosis of the HCT-8 epithelial cell line induced by the parasite *Giardia intestinalis*. FEMS Immunol Med Microbiol. 2007;51: 302–309. doi: 10.1111/j.1574-695X.2007.00304.x 17714487 10.1111/j.1574-695X.2007.00304.x

[112] Ono SJ, Nakamura T, Miyazaki D, Ohbayashi M, Dawson M, Toda M, et al Chemokines: roles in leukocyte development, trafficking, and effector function. J Allergy Clin Immunol. Garland, New York; 2003;111: 1185–99; quiz 1200. doi: 10.1067/MAI.2003.1594 10.1067/mai.2003.1594 12789214

[113] Lee H-YH-Y, Hyung S, Lee NY, Yong T-ST-S, Han S-HS-H, PARK S-JS-J. Excretory-secretory products of *Giardia lamblia* induce interleukin-8 production in human colonic cells via activation of p38, ERK1/2, NF-κB and AP-1. Parasite Immunol. 2012;34: 183–98. doi: 10.1111/j.1365-3024.2012.01354.x 22224945 10.1111/j.1365-3024.2012.01354.x

[114] Patterson KI, Brummer T, O’brien PM, Daly RJ. Dual-specificity phosphatases: critical regulators with diverse cellular targets. Biochem J. 2009;418 Available: http://www.biochemj.org/content/418/3/475.long 10.1042/bj20082234 19228121

[115] Guan KL, Butch E. Isolation and characterization of a novel dual specific phosphatase, HVH2, which selectively dephosphorylates the mitogen-activated protein kinase. J Biol Chem. American Society for Biochemistry and Molecular Biology; 1995;270: 7197–203. doi: 10.1074/JBC.270.13.7197 10.1074/jbc.270.13.7197 7535768

[116] Ishibashi T, Bottaro DP, Michieli P, Kelley CA, Aaronson SA. A novel dual specificity phosphatase induced by serum stimulation and heat shock. J Biol Chem. 1994;269: 29897–902. Available: http://www.ncbi.nlm.nih.gov/pubmed/7961985 7961985

[117] Clark AR, Dean JLE. The control of inflammation via the phosphorylation and dephosphorylation of tristetraprolin: a tale of two phosphatases. Biochem Soc Trans. 2016;44: 1321–1337. doi: 10.1042/BST20160166 27911715 10.1042/BST20160166 PMC5095909

[118] Tiedje C, Diaz-Muñoz MD, Trulley P, Ahlfors H, Laaß K, Blackshear PJ, et al The RNA-binding protein TTP is a global post-transcriptional regulator of feedback control in inflammation. Nucleic Acids Res. 2016;44: 7418–40. doi: 10.1093/nar/gkw474 27220464 10.1093/nar/gkw474 PMC5009735

[119] In JG, Foulke-Abel J, Estes MK, Zachos NC, Kovbasnjuk O, Donowitz M. Human mini-guts: new insights into intestinal physiology and host—pathogen interactions. Nat Rev Gastroenterol Hepatol. Nature Research; 2016;13: 633–642. doi: 10.1038/nrgastro.2016.142 27677718 10.1038/nrgastro.2016.142 PMC5079760

