# Supplementary figures and images for: Characterization of the Giardia intestinalis secretome during interaction with human intestinal epithelial cells: The impact on host cells
Source: PLoS Negl Trop Dis. 2017 Dec 11;11(12):e0006120. doi: 10.1371/journal.pntd.0006120 (PMC5739509; doi:10.1371/journal.pntd.0006120)

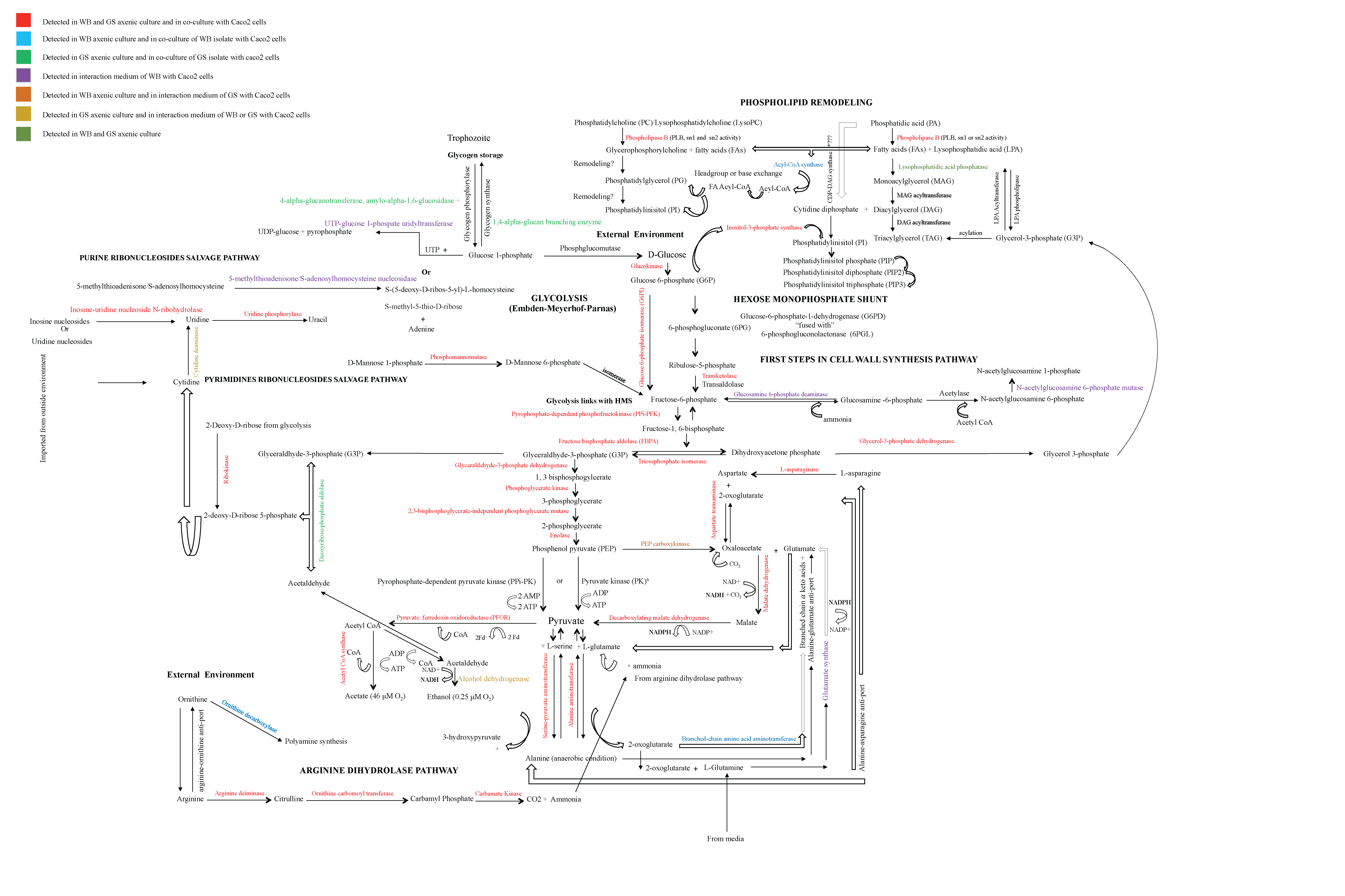

Supplement: S1 Fig — (TIFF) [file pntd.0006120.s001.tiff]

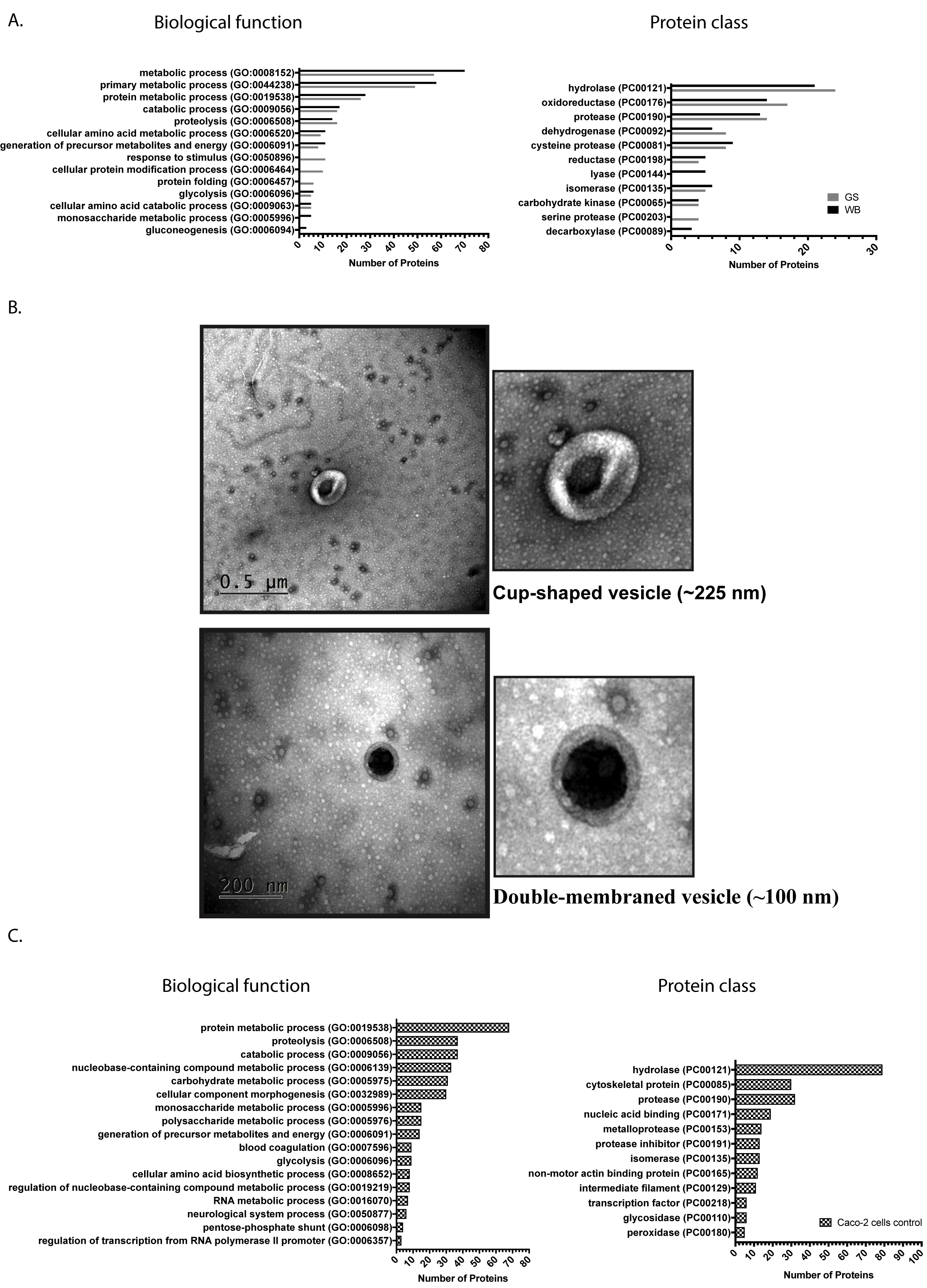

Supplement: S2 Fig — (A) GO term analyses of WB and GS trophozoites in axenic culture. (B) Electron micrographs of Giardia extracellular vesicles. (C) GO term analyses of differentiated Caco-2 cell secretome in the absence of parasite. (TIF) [file pntd.0006120.s002.tif]

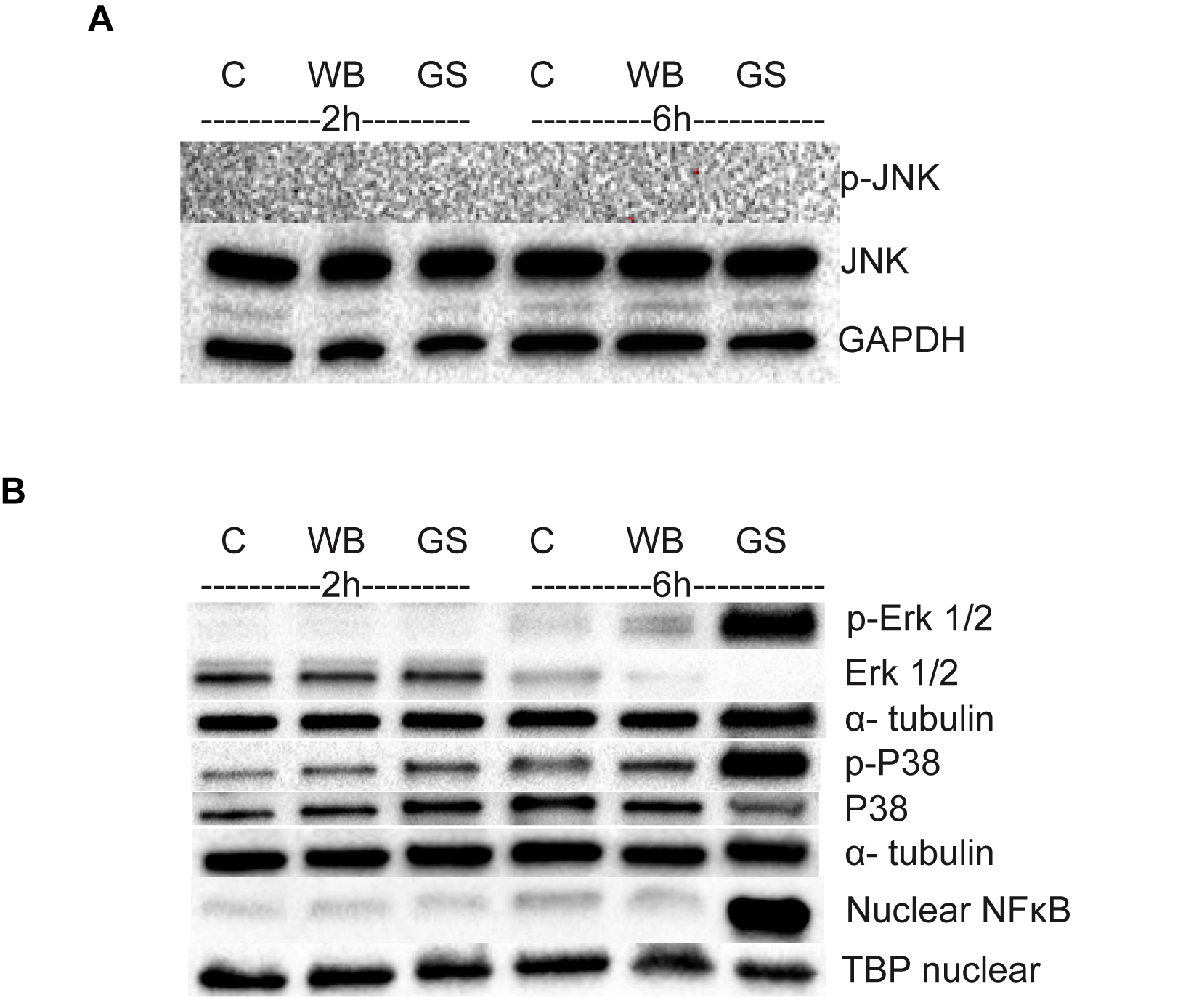

Supplement: S3 Fig — (A) Western blot analyses of differentiated Caco-2 cells exposed to G. intestinalis ESPs through a Transwell insert for 2h or 6h in the absence of inflammatory stimuli. Blots were probed for the phosphorylated/non-phosphorylated forms of JNK. (B) Western blot analyses of differentiated Caco-2 cells exposed to G. intestinalis ESPs collected in axenic culture (i.e. in the absence of intestinal epithelial cells). Blots were probed for total proteins (phosphorylated/non-phosphorylated forms) of mitogen activated proteins kinases (MAPKs, ERK1/2 and P38) and nuclear factor kappa beta (NFκB). (TIF) [file pntd.0006120.s003.tif]
